# RANBP3 promotes mitophagy through the CCAR2/SIRT1 pathway to alleviate pyroptosis in macrophages in TBTB

**DOI:** 10.1038/s41598-026-55696-2

**Published:** 2026-06-11

**Authors:** Lei Zhou, Lin-Zi Luo, Zhi-Bin Lu, Hai-Long Luo, Dan Feng, Limei Gong, Yang-Bao Xiao, Li Luo

**Affiliations:** Endoscopy Center, Hunan Institute for Tuberculosis Control (Hunan Chest Hospital), Changsha, 410013 China

**Keywords:** TBTB, Macrophage pyroptosis, Mitophagy, CCAR2/SIRT1 pathway, RANBP3, Cell biology, Diseases, Immunology, Microbiology, Molecular biology, Pathogenesis

## Abstract

Mycobacterium tuberculosis infection of the trachea causes tracheobronchial tuberculosis (TBTB), a chronic inflammatory illness, and its pathological features include macrophage pyroptosis. RanBP3, as a potential regulator, participates in cell stress, but its specific mechanism of action in TBTB has not been clarified. The mouse and bone marrow-derived macrophages (BMDMs) models were constructed by infecting with *M. smegmatis*. RanBP3 protein level and the levels of pyroptosis-related proteins were measured by Western blotting (WB). The levels of inflammatory factors were measured by ELISA. Pearson’s correlation analysis was employed to evaluate their correlation with RanBP3, and the interaction of RanBP3/CCAR2/SIRT1 was verified by Co-immunoprecipitation (Co-IP). The detection of macrophage pyroptosis and mitophagy was done using flow cytometry, and mitophagy was further assessed by transmission electron microscopy. Bacterial survival in BMDMs was evaluated by colony-forming unit assays. Hematoxylin and eosin (H&E) and Masson staining were used to assess lung tissue damage. RanBP3 was down-regulated in TBTB mucosa. At the cellular level, RanBP3 overexpression alleviated BMDM’s pyroptosis by promoting mitophagy and reduced intracellular bacterial survival, whereas RanBP3 knockdown exacerbated pyroptosis and increased bacterial survival. Mechanistically, RanBP3 competitively binds to CCAR2, disrupts the CCAR2-SIRT1 interaction, and promotes SIRT1 release, thereby activating mitophagy. At the animal level, RanBP3 overexpression promoted mitophagy, alleviated macrophage pyroptosis, and alleviated TBTB, but the SIRT1 inhibitor reversed these effects. RanBP3 mediates the release of SIRT1 through the CCAR2-SIRT1 axis, thereby activating mitophagy and inhibiting pyroptosis, providing a new target for TBTB treatment.

**Supplementary Information:**

The online version contains supplementary material available at 10.1038/s41598-026-55696-2.

## Introduction

A specific form of tuberculosis, tracheobronchial tuberculosis (TBTB), is caused by an infection of the trachea, bronchial mucosa, submucosa, smooth muscle, cartilage, and outer membrane by Mycobacterium tuberculosis (Mtb)^1–3^. Without prompt and effective treatment, TBTB can result in tracheal and bronchial narrowing and obstruction, leading to respiratory dysfunction, secondary infections, and severe complications such as atelectasis and hemoptysis^4–6^. According to the World Health Organization’s 2022 Global Tuberculosis Report, there were an estimated 10.6 million tuberculosis cases worldwide in 2021^7^. Despite this, traditional treatment approaches for TBTB, including interventional therapy, radiation therapy, local drug therapy, and surgery, have limitations in terms of clinical diagnosis and treatment efficacy^8,9^. Therefore, new treatment strategies and targets are essential for addressing tracheobronchial tuberculosis effectively.

Mtb is an intracellular parasite that thrives within macrophages, triggering excessive inflammatory responses and aggravating tissue damage^10,11^. Pyroptosis serves as a vital facet of the host’s innate immune system in response to Mtb infection. This process acts as an effective antibacterial mechanism, exacerbating inflammatory reactions^12–14^. According to studies, Mtb can trigger pyroptosis in a Caspase-1 / NOD-like receptor family pyrin domain containing protein 3 (NLRP3) / Gasdermin D (GSDMD) -dependent way and increase the production of pro-inflammatory cytokines in macrophages by activating the nuclear factor κB (NF-κB) signal^15,16^. Macrophage pyroptosis emits a lot of Interleukin-1β (IL-1β) and Interleukin-32γ (IL-32γ), and Caspase-1 and IL-32γ work together to let Mtb escape to neighboring cells^17,18^. Therefore, controlling macrophage pyroptosis to maintain a balance between immune defense and pathological damage could potentially alleviate TBTB symptoms.

Pyroptosis and mitophagy are distinct processes of programmed cell death that can be activated simultaneously by various stimuli and contribute to the onset or progression of a variety of diseases^19,20^. There exists a complex interplay between the two processes. Previous studies have shown that mitophagy can alleviate pyroptosis by removing damaged mitochondria, reducing reactive oxygen species (ROS) production, and inhibiting NLRP3 inflammasome activation^20–22^. Therefore, in-depth elucidation of the regulatory network between mitophagy and pyroptosis and identification of its key molecules may yield novel therapeutic approaches for reducing the pathological harm caused by TBTB.

Silent Information Regulator 1 (SIRT1) is an NAD-dependent deacetylase. Increased SIRT1 activity promotes the expression of autophagy-related genes such as Phosphatase and tensin homologue deleted on chromosome 10 (PTEN) induced putative kinase 1 (PINK1), Parkin, and Beclin1 (BECN1)^23,24^. Upregulation of the SIRT1 signaling pathway can inhibit macrophage pyroptosis by affecting the expression of pyroptosis-related factors such as NLRP3 and GSDMD^25–27^. As a transcriptional regulator, Cell Cycle and Apoptosis Regulator 2 (CCAR2) inhibits the function of SIRT1 by interacting with its catalytic site. Disrupting the interaction between CCAR2 and SIRT1 promotes the expression of SIRT1 and the activation of autophagy^28^. Previous studies have shown that tumor suppressor factors may be associated with pyroptosis. For example, the tumor suppressor gene Dopamine Receptor D2 (DRD2) can limit NF-κB signaling and promote pyroptosis in breast cancer cells^29^. Gasdermin E (GSDME) from the Gasdermin protein family is considered a tumor suppressor gene involved in the development of pyroptosis^30^. Like DRD2 and GSDME, RanBP3 is regarded as a tumor suppressor gene and has a role in senescence, apoptosis, and cell division^23,24^. We speculate that the tumor suppressor gene RanBP3 may also influence pyroptosis; however, more research is required to determine whether RanBP3 regulates macrophage pyroptosis and the underlying mechanisms.

Investigating RanBP3 expression patterns in TBTB and its impact on the CCAR2-SIRT1 pathway is the aim of this study. The main objective is to ascertain whether RanBP3 controls pyroptosis and mitophagy via the CCAR2-SIRT1 axis. In doing so, our study aims to offer new insights and pinpoint possible therapeutic targets for the successful clinical treatment of tuberculosis.

## Materials and methods

### Clinical sample collection

Clinical samples were collected from a group of ten patients diagnosed with TBTB, who had granulation tissue (bronchial mucosa) removed for analysis. A comparison group of six patients with aspergilloma underwent removal of normal bronchial mucosa from the lung resection stump. Approval for this study was obtained from the Ethics Review Committee of Hunan Institute for Tuberculosis Control (Hunan Chest Hospital) (Approval No.: LS2024052901), and all patients or their legal guardians provided informed consent for sample collection. The research was conducted according to the World Medical Association Declaration of Helsinki. Standard anti-tuberculosis medication was administered to all TBTB patients, while the control group comprised healthy individuals with no history of tuberculosis or serious systemic illnesses. Each participant underwent a comprehensive clinical examination to assess their current health status.

### Bacterial strains and culture conditions

Given the high biosafety requirements for Mtb operation and the focus of this study on the early mechanism exploration, we used non-pathogenic *Mycobacterium smegmatis* (*M. smegmatis*) as an alternative infection model for Mtb. *M. smegmatis* strains were cultured on Middlebrook 7H10 agar supplemented with 10% oleic acid-albumin-dextrose-catalase (OADC) enrichment. Colonies cultured for 1 week and 4 weeks were respectively inoculated into Middlebrook 7H9 liquid medium containing 10% ADC or Sauton medium with different components to initiate subsequent liquid culture^31^.

### Animals

Male C57BL/6J mice, 9–12 weeks old, 20–25 g, were purchased from Hunan SJA Laboratory Animal Co., Ltd. After acclimatization for one week, the animals were randomly divided into five groups: Control; TBTB; TBTB + oe-NC; TBTB + oe-RanBP3; and TBTB + oe-RanBP3 + EX-527. The TBTB group was infected with *M. smegmatis* strain by nasal inoculation of 50 μL bacterial suspension (5.4 × 10^7^ ± 0.9 CFU)^32^. Before infection, the TBTB + oe-NC and TBTB + oe-RanBP3 groups were injected with oe-NC and oe-RanBP3 lentivirus through the tail vein, and each mouse was injected with 100 µL 1 × 10^8^ TU/ml lentivirus, and the intervention was performed once every two weeks. Prior to aerosol intervention, the TBTB + oe-RanBP3 + EX-527 group was given a viral injection. EX-527 (5 mg/kg, HY-15452, MCE, USA) was then administered intraperitoneally as an intervention. After 16 weeks, mice were euthanized by cervical dislocation after being anesthetized with isoflurane (PHR2874, Sigma-Aldrich, USA) until they lost consciousness, and samples of the mice’s lung tissue were taken for further research. All experimental procedures and animal handling were performed with the approval of the Animal Care and Use Committee of the Hunan Institute for Tuberculosis Control (Hunan Chest Hospital) (Approval No. LS2024052901), in accordance with the National Institutes of Health Guide for the Care and Use of Laboratory Animals, and studies involving laboratory animals follow the ARRIVE guidelines.

### Hematoxylin and eosin (HE) and masson staining

HE staining: tissue sections were first baked at 60 °C for 1–2 h, dewaxed with xylene and graded ethanol, then stained with hematoxylin-eosin, counterstained with blue, and finally dehydrated with alcohol, transparentized with xylene, and sealed with neutral gum for microscopic observation.

Masson staining: tissue sections were baked at 60 °C for 1–2 h, dewaxed and hydrated with xylene and graded ethanol, and then nuclear staining and blue return, cytoplasmic staining, color separation, and restaining were performed in sequence. Finally, the sections were blown dry, transparentized with xylene, and sealed for observation.

### Cell culture

Bone marrow (BM) cells were isolated from the tibia and femur of male C57BL/6J mice^33,34^ and cultured in low-glucose DMEM/F-12 medium (iCell-0005, iCell-BioTech, USA) supplemented with 10% fetal bovine serum (10099141, Gibco, USA), 1% P/S (C0222, Beyotime, China), and 10 ng/ml M-CSF (M9170, Sigma, USA). The cell suspension was then plated in T25 culture flasks and cultured at 37 °C in 5% CO_2_. Mouse bone marrow-derived macrophages (BMDMs) were identified by flow cytometry using anti-mouse CD11b (APC) antibody (17-011282, eBioscience, USA) and anti-mouse F4/80 (FITC) antibody (11-4801-82, eBioscience, USA). All BMDMs were tested for mycoplasma contamination to any experimental procedures and confirmed to be negative.

### Cell treatment

BMDMs were transfected with various transfection reagents for 24 h, including an overexpression plasmid (oe-RanBP3), a negative control plasmid (oe-NC), a silencing transfection plasmid (si-CCAR2), a silencing transfection plasmid (si-SIRT1), and a negative control transfection plasmid (si-NC). The specific sequences used were as follows: CCAR2 siRNA sequence: 5’-GTGGTAGATGAAGAAGTCTTT-3’; SIRT1 siRNA sequence: 5’-GGCTTTCATTCCTGTGAAAGT-3’; NC siRNA sequence: 5’-TGCCGACGGGTACCGGGCAAC-3’. Cell transfection was conducted using Lipofectamine 2000 (11668019, Invitrogen, USA) as per the manufacturer’s instructions. Following transfection, BMDMs were treated with the autophagy inhibitor Mdivi-1 (HY-15886, MCE, USA), Oroxylin A (HY-N0560, MCE, USA) for 24 h, or the SIRT1 enzyme activity inhibitor EX-527 (HY-15452, MCE, USA) for 1 h. Subsequently, BMDMs were infected with the *M. smegmatis* strain at a multiplicity of infection (MOI) of 2 for 4 h.

### Western blot (WB)

Tissue/cells were lysed using RIPA lysis buffer (AWB0136, Abiowell, China), and proteins were extracted. The antibodies used are listed in Table 1. Color development was performed using ECL chemiluminescent solution (AWB0005, Abiowell, China), followed by exposure using a gel imaging system (ChemiScope 6100, Clinx Science Instruments, China).

**Table 1:** Antibody information.

Name	Item number	Dilution ratio	Company
RanBP3	27168-1-AP	1:1000	Proteintech (USA)
Caspase 1	ab207802	1:1000	Abcam (UK)
NLRP3	19771-1-AP	1:800	Proteintech
GSDMD	66387-1-Ig	1:10000	Proteintech
LC3	14600-1-AP	1:3000	Proteintech
Beclin1	AWA12678	1:1000	Abiowell (China)
Pink1	ab216144	1:1000	Abcam
Parkin	AWA41194	1:1000	Abiowell
CCAR2	22638-1-AP	1:10000	roteintech
SIRT1	AWA03347	1:1000	Abiowell
β-actin	66009-1-Ig	1:5000	Proteintech
HRP goat anti-mouse IgG	SA00001-1	1:5000	Proteintech
HRP goat anti-rabbit IgG	SA00001-2	1:6000	Proteintech

### Quantitative real-time reverse transcription polymerase chain reaction (qRT-PCR)

Total RNA from BMDMs was extracted using TRIzol reagent (15596026, Thermo, USA) and reverse transcribed into cDNA using the HiFiScript cDNA Synthesis Kit (CW2569, CWbio, China). qRT-PCR was performed using UltraSYBR Mixture (CW2601, CWbio, China) on a PIKOREAL96 system (Thermo). The primer sequences used are as follows: RanBP3 (F: AGTAAAATGGCGGACCTGGC, R: GACTCCGGCAAGTCTCTGTG), CCAR2 (F: CAACGTCCTTTGCACCCCTA, R: CCTTTTCCTCCCGTCTTGCT), SIRT1 (F: TTCTATACCCCATGAAGTGCCTC, R: CACCACCTAGCCTATGACACA), and β-actin (F: ACATCCGTAAAGACCTCTATGCC, R: TACTCCTGCTTGCTGATCCAC). The relative gene expression levels were calculated using the 2^−ΔΔCt^ method and normalized to β-actin as the internal control.

### Colony-forming unit (CFU) assay^35^

BMDMs or THP-1 cells differentiated with Phorbol 12-myristate 13-acetate (0.1 µg/mL, 48 h) were seeded in 12-well plates at a density of 1 × 10^6^ cells per well. Cells were infected with Ms_EsxN or Ms_pALACE strains with MOI of 10. After 4 h of infection, the supernatant was discarded, and the cells were washed three times with sterile PBS to remove unbound bacteria. Fresh medium containing 10% FBS and gentamicin (final concentration 100 µg/mL) was then added to continue culturing and eliminate extracellular bacteria. Cells were collected at different time points and lyse them with PBS containing 0.025% SDS. After performing a serial dilution of the lysate, it was spread onto 7H10 agar plates, incubated at 37 °C for 3–4 days, and the colony-forming units were counted.

### Transmission electron microscope (TEM)

BMDMs from each group were collected, prefixed with glutaraldehyde (AWI0097, Abiowell, China), and then fixed with 1% osmic acid (AWI0136, Abiowell, China). After gradual dehydration with acetone, the samples were infiltrated and embedded with Epon-812 embedding medium (GS02659, Beijing Zhongjing Keyi Technology Co., Ltd., China). The ultrathin microtome (Leica UC-7, Leica, China) was used to cut 60–90 nm sections, which were then collected onto copper grids (BZ11262a, Zhongjingkeyi Technology Co., Ltd.) and stained at room temperature with uranyl acetate (GZ02625, Zhongjingkeyi Technology Co., Ltd.) and lead citrate (GA10701-1, Zhongjingkeyi Technology Co., Ltd.) sequentially. Observation and image acquisition were performed using a transmission electron microscope (JEM-1400FLASH, JEOL, Japan). Autophagosomes were identified as double- or multi-membraned vacuolar structures containing cytoplasmic components, including mitochondria.

### Enzyme-linked immunosorbent assay (ELISA) and biochemical examination

IL-1β (KE10003, Proteintech, USA) and Interleukin-18 (IL-18) (CSB-E04609m, Cusabio, China) levels were detected using ELISA kits. Standard samples were diluted in a gradient, and both samples and standards were added to the microplate. The following steps were performed sequentially: primary antibody incubation (37 °C for 2 h), biotin-labeled secondary antibody incubation (37 °C for 1 h), HRP-streptavidin reaction (37 °C for 40 min to 1 h), and TMB color development. The OD value was measured at 450 nm.

Lactate dehydrogenase (LDH) levels were measured using the lactate dehydrogenase assay kit (A020-2, NJJC Bio, China). The supernatant of cell/tissue samples after homogenization and centrifugation was taken. The activity of lactate dehydrogenase was determined via an enzymatic reaction, and the unit enzyme activity was calculated according to the standard formula.

### Flow cytometry

Pyroptosis assay kit (ab219935, Abcam, UK), JC-1 assay kit (C2006, Beyotime, China), and MitoSOX Red (S0061S, Beyotime, China) were used to detect pyroptosis, mitochondrial function, and macrophage phenotype. Pyroptosis assay: the staining solution (300 µL buffer + 2 µL FAM-YVAD-FMK) was prepared in the dark, the samples were stained for 1 h, and after washing, then counterstained with PI for 10 min, and finally, the machine was tested. JC-1 assay: cells were co-incubated with JC-1 working solution at 37 °C for 20 min, centrifuged at 4 °C, washed twice with 1X buffer, and analyzed by flow cytometry. MitoSOX fluorescence acquisition: 1 µM working solution was used to cover cells, incubated at 37 °C for 10 min, washed three times with serum-free medium, and then analyzed by flow cytometry. The above operations are performed in the dark.

### Co-immunoprecipitation (Co-IP)

Immunoprecipitation was used to validate protein interactions: after cell lysis, CCAR2 and RanBP3 antibodies were left to incubate for the entire night at 4 °C. Following the complexes’ capture by Protein A/G beads and subsequent washing, WB analysis was used to determine the interactions involving RanBP3, CCAR2, and SIRT1, and the binding affinity was assessed by calculating the IP/Input ratio after densitometric quantification.

### Immunofluorescence (IF)

The double-label multiplex immunofluorescence assay kit TSA-520, TSA-620 (AWI0693a, Abiowell, China) was used to detect the expression of CCAR2 and SIRT1 in BMDMs: After permeabilization and blocking, the cells were incubated with primary antibodies against CCAR2 and SIRT1 (overnight at 4 °C/37°C for 2 h), followed by incubation with HRP-conjugated secondary antibodies (37 °C for 30 min) and labeled with different fluorescent dyes. The nuclei were stained with DAPI, and the slides were mounted for observation.

### Statistical analysis

All experimental data are presented as mean ± standard deviation (SD). Each experiment was performed with at least three biological replicates. Statistical analysis was performed using GraphPad Prism 8 software. Comparisons between two groups were conducted using Student’s t-test, while comparisons among multiple groups were performed using one-way analysis of variance (ANOVA) combined with Tukey’s post hoc test. The significance level was set at *P* < 0.05.

## Results

### RanBP3 expression in the airway mucosal tissue of TBTB patients is negatively correlated with pyroptosis

**Fig. 1:**
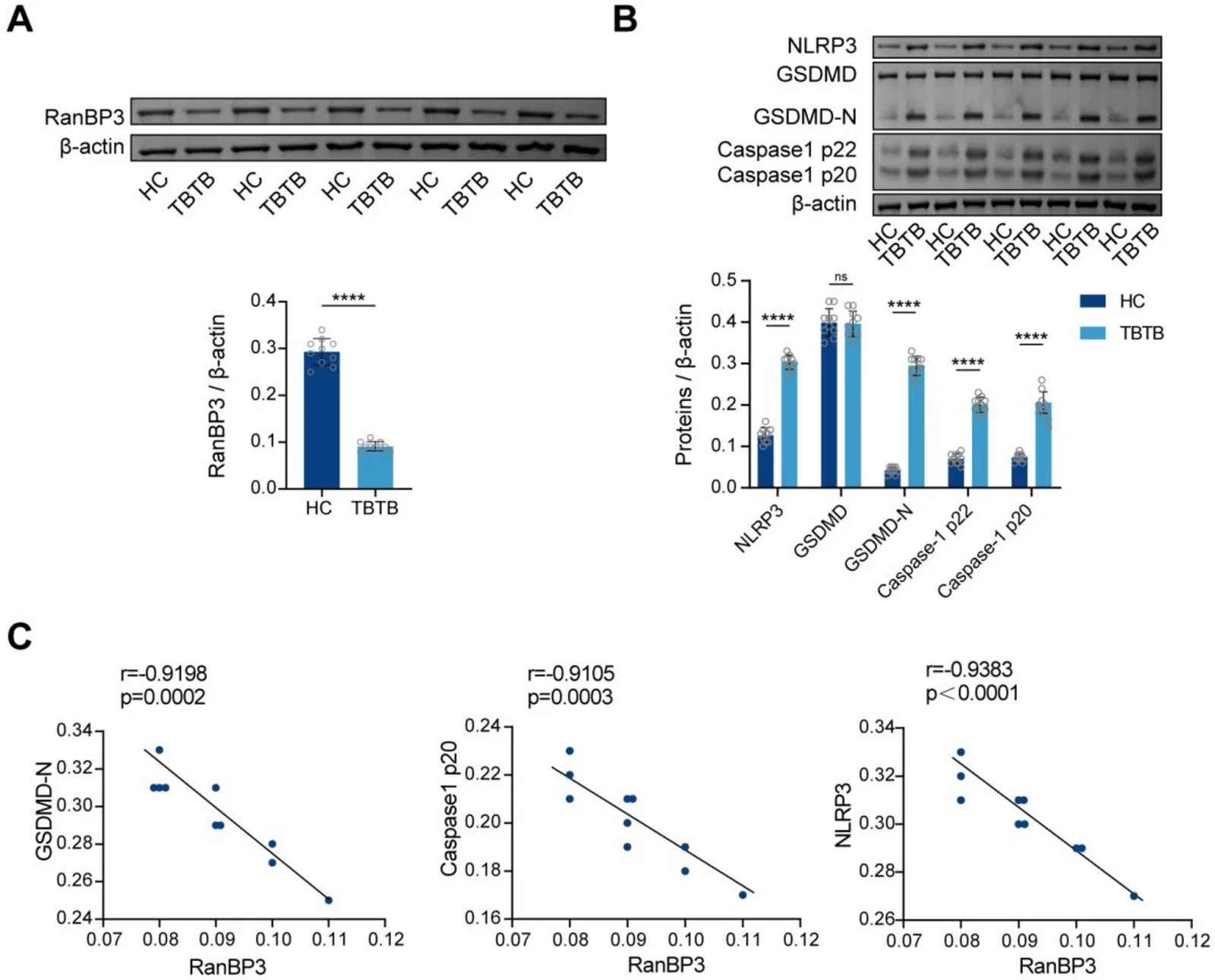
RanBP3 expression in the airway mucosal tissue of TBTB patients is negatively correlated with pyroptosis. (**A**) RanBP3 protein level was detected in WB. (**B**) WB analysis was conducted to identify the protein levels of NLRP3, Caspase-1 p22/p20, GSDMD, and GSDMD-N, factors associated with pyroptosis, in the tissues. (**C**) The link between RanBP3 and NLRP3, GSDMD-N, and Caspase-1 p20 was investigated using Pearson’s analysis. n = 10. ****P < 0.0001.

RanBP3 is a potential therapeutic target for various cancers^36–38^, and current research indicates that RanBP3 is primarily associated with cell proliferation^39,40^. However, the protein level of RanBP3 in TBTB and its role in pyroptosis remain unknown. First, we used WB analysis to detect the protein level of RanBP3 in the lesion mucosa of patients with bronchial tuberculosis and in normal airway mucosa tissue. The results showed that compared with normal airway mucosa tissue, the protein level of RanBP3 was downregulated in the lesion mucosa of patients with bronchial tuberculosis (Fig. 1A). Next, we further investigated the effect of RanBP3 on pyroptosis. WB analysis was performed to detect the protein levels of pyroptosis-related factors GSDMD, GSDMD-N, Caspase-1 p22/p20, and NLRP3 in the tissues. The results showed that the GSDMD protein level was similar between the TBTB group and the Control group. However, compared with the Control group, GSDMD-N, Caspase-1 p22/p20, and NLRP3 protein levels were increased in the TBTB group (Fig. 1B). Lastly, RanBP3 was found to be negatively correlated with GSDMD-N, Caspase-1 p20, and NLRP3 protein levels by Pearson’s analysis (Fig. 1C). In summary, the above results indicate that RanBP3 expression is inversely associated with pyroptosis in the airway mucosal tissue of TBTB patients.

### RanBP3 regulates pyroptosis in macrophages infected with *M. smegmatis*

**Fig. 2:**
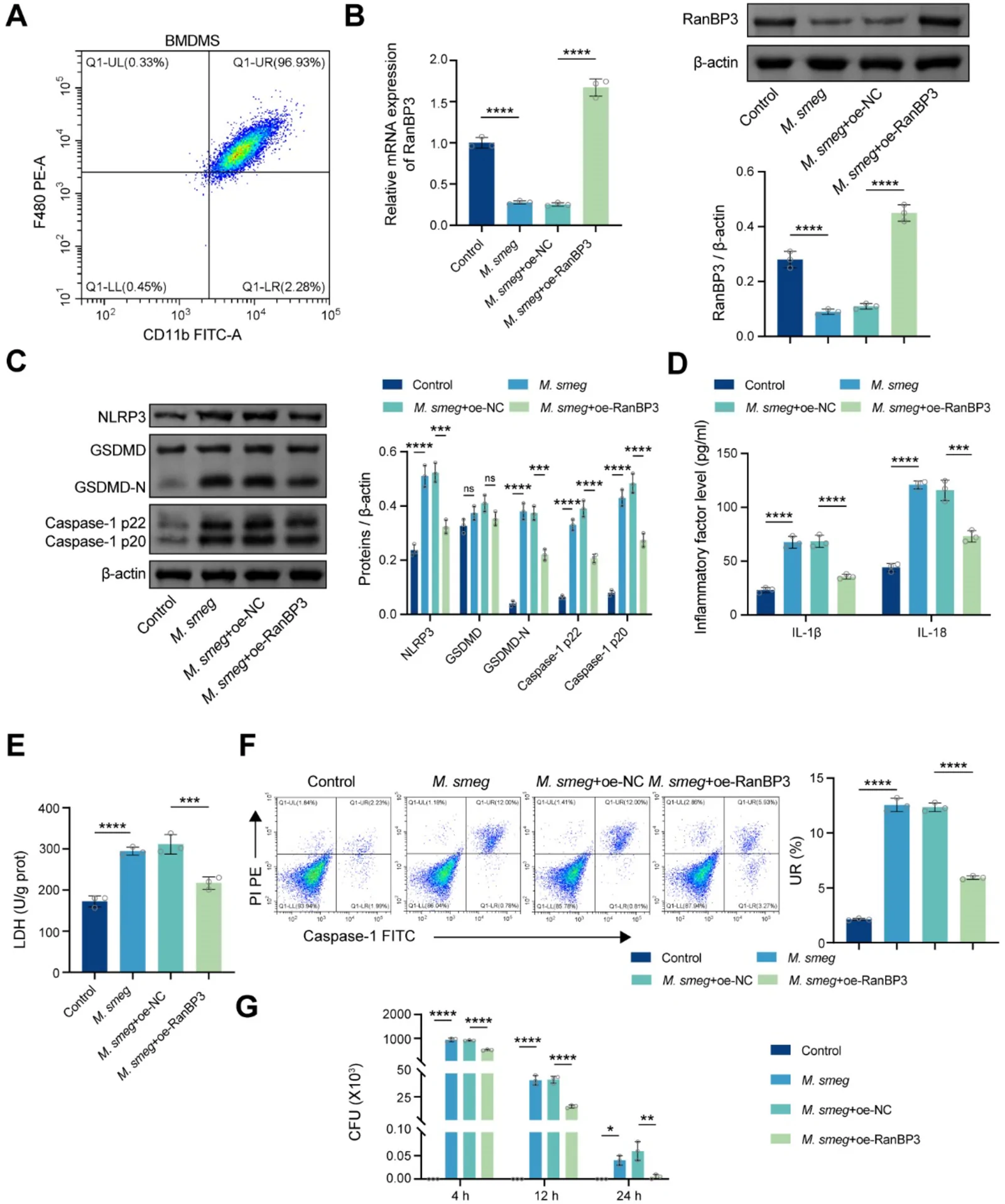

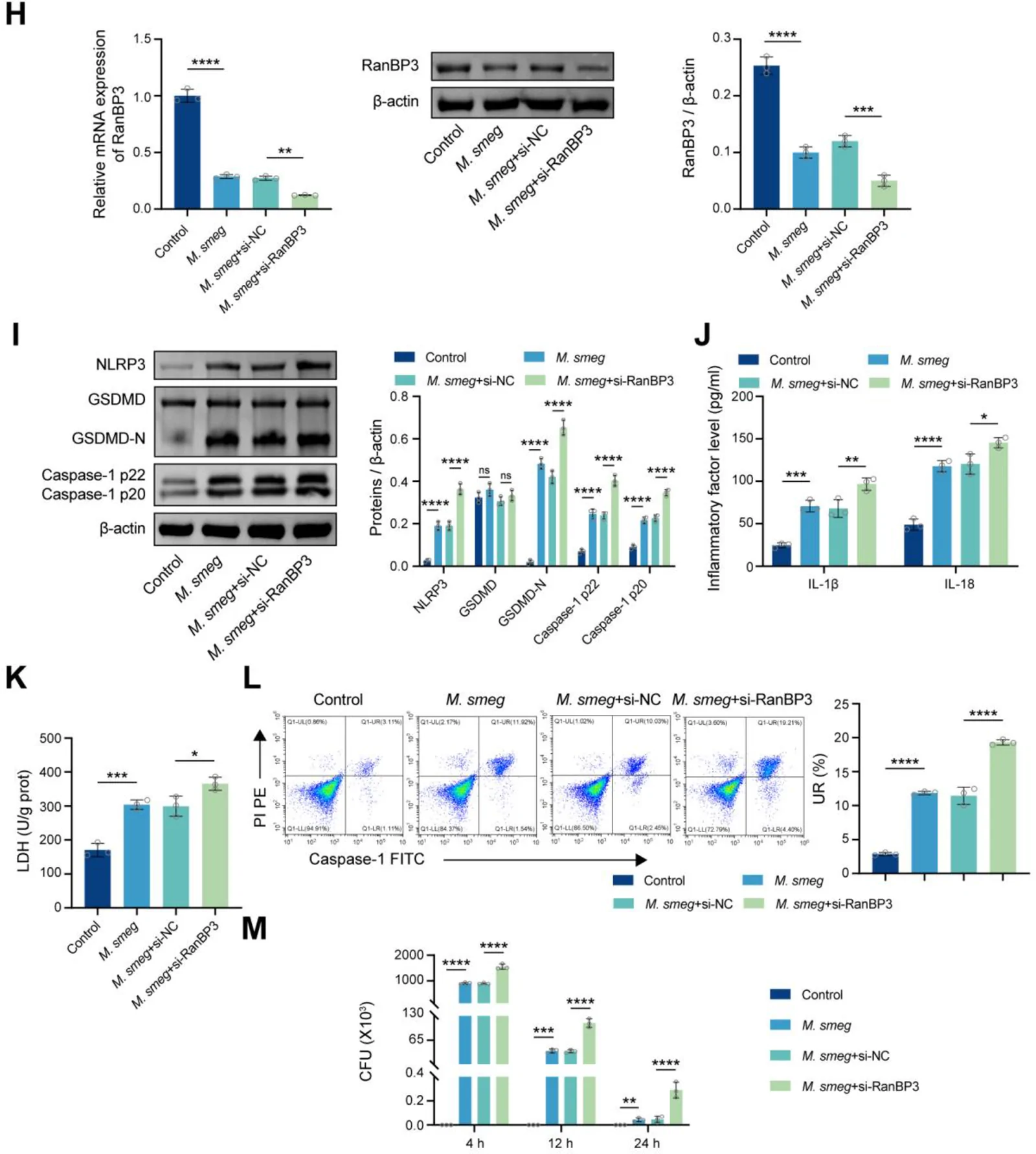
RanBP3 regulates pyroptosis in macrophages infected with *M. smegmatis*. (**A**) BMDMs were isolated from C57BL/6J mice and stained with CD11b and F4/80 antibodies, followed by flow cytometry analysis to assess macrophage purity. BMDMs were transfected with overexpression plasmids or silencing plasmids for 24 h, followed by infection with *M. smegmatis* (MOI = 2) for 4 h. Based on the above treatments, the following assays were performed. (**B**,** H**) qRT-PCR and WB detection of RanBP3 mRNA and protein levels in BMDMs. (**C**,** I**) WB detection of GSDMD, GSDMD-N, Caspase-1 p22/p20, and NLRP3 protein levels in BMDMs. (**D**,** J**) ELISA detection of IL-1β and IL-18 levels in cell supernatants. (**E**,** K**) ELISA detection of LDH levels in cell supernatants. (**F**,** L**) Flow cytometry detection of pyroptosis. (**G**,** M**) CFU detection assesses the survival rate of *M. smeg* in BMDMs. *M. smeg*: cells infected with *M. smegmatis*. n = 3. *P < 0.05; **P < 0.01; ***P < 0.001; ****P < 0.0001, ns stands for no significant difference.

To investigate the impact of RanBP3 overexpression on pyroptosis in *M. smegmatis*-infected BMDMs, we further examined pyroptosis-related markers in BMDMs isolated after RanBP3 overexpression intervention. Initially, the percentage of CD11b and F4/80 double-positive cells in the acquired BMDMs approached 90%, according to flow cytometry analysis, suggesting a very pure macrophage population appropriate for further experimental research (Fig. 2A). To verify the reliability of the overexpression model, we first assessed the transfection efficiency of RanBP3. The results of qRT-PCR and WB show that compared to the oe-NC group, the mRNA and protein levels of RanBP3 in the oe-RanBP3 group were significantly increased (Figure S1A), indicating that the overexpression model was successfully constructed. After transfection with RanBP3 overexpression plasmids, the mRNA and protein levels of RanBP3 in BMDMs were significantly increased (Fig. 2B). Compared to the Control group, the *M. smeg* group exhibited upregulated protein levels of GSDMD-N, Caspase-1 p22/p20, and NLRP3. Conversely, RanBP3 overexpression resulted in downregulation of these pyroptosis-related markers when compared to the *M. smeg* + oe-NC group; notably, the protein level of GSDMD remained unaffected by *M. smegmatis* infection or RanBP3 overexpression (Fig. 2C). Furthermore, ELISA findings indicated that release levels of IL-1β, IL-18, and LDH were elevated in the *M. smeg* group in comparison to the Control group. In contrast, RanBP3 overexpression led to the inhibition of IL-1β, IL-18, and LDH expression levels when compared to the *M. smeg* + oe-NC group (Fig. 2D and E). Flow cytometry results showed that the proportion of pyroptotic cells increased in the *M. smeg* group compared to the Control group. Compared to the *M. smeg* + oe-NC group, the proportion of pyroptotic cells decreased in the RanBP3 overexpression group (Fig. 2F). Furthermore, the CFU assay results showed that the bacterial survival rate within macrophages in the *M. smeg* group increased significantly, whereas it decreased after RanBP3 overexpression (Fig. 2G).

To further confirm the role of RanBP3 in macrophage pyroptosis, we used siRNA to knock down RanBP3 for validation. The results of qRT-PCR and WB showed that compared to the si-NC group, the mRNA and protein levels of RanBP3 in the si-RanBP3 groups were significantly reduced, with si-RanBP3#3 showing the highest knockdown efficiency (Figure S1B). After transfection with RanBP3 silencing plasmids, the mRNA and protein levels of RanBP3 in BMDMs were significantly decreased (Fig. 2H). Compared to the Control group, the protein levels of GSDMD-N, Caspase-1 p22/p20, and NLRP3 were upregulated in the *M. smeg* group. Compared to the *M. smeg* + si-NC group, the protein levels of these pyroptosis-related markers were further elevated in the RanBP3 knockdown group; there were no significant changes in the protein level of GSDMD among the groups (Fig. 2I). The ELISA results showed that, compared to the *M. smeg* + si-NC group, the release levels of IL-1β, IL-18, and LDH were further elevated in the RanBP3 knockdown group (Fig. 2J and K). The flow cytometry results showed that compared to the *M. smeg* + si-NC group, the proportion of pyroptotic cells in the RanBP3 knockdown group further increased (Fig. 2L). The CFU assay results showed that compared to the *M. smeg* + si-NC group, the bacterial survival rate within macrophages was significantly higher in the RanBP3 knockdown group (Fig. 2M).

The above results indicate that RanBP3 overexpression can alleviate *M. smegmatis*-induced macrophage pyroptosis and reduce bacterial load, while RanBP3 knockdown exacerbates pyroptosis, confirming that RanBP3 plays a protective role in infection.

### Overexpression of RanBP3 alleviates macrophage pyroptosis and is associated with enhanced mitophagy

**Fig. 3:**
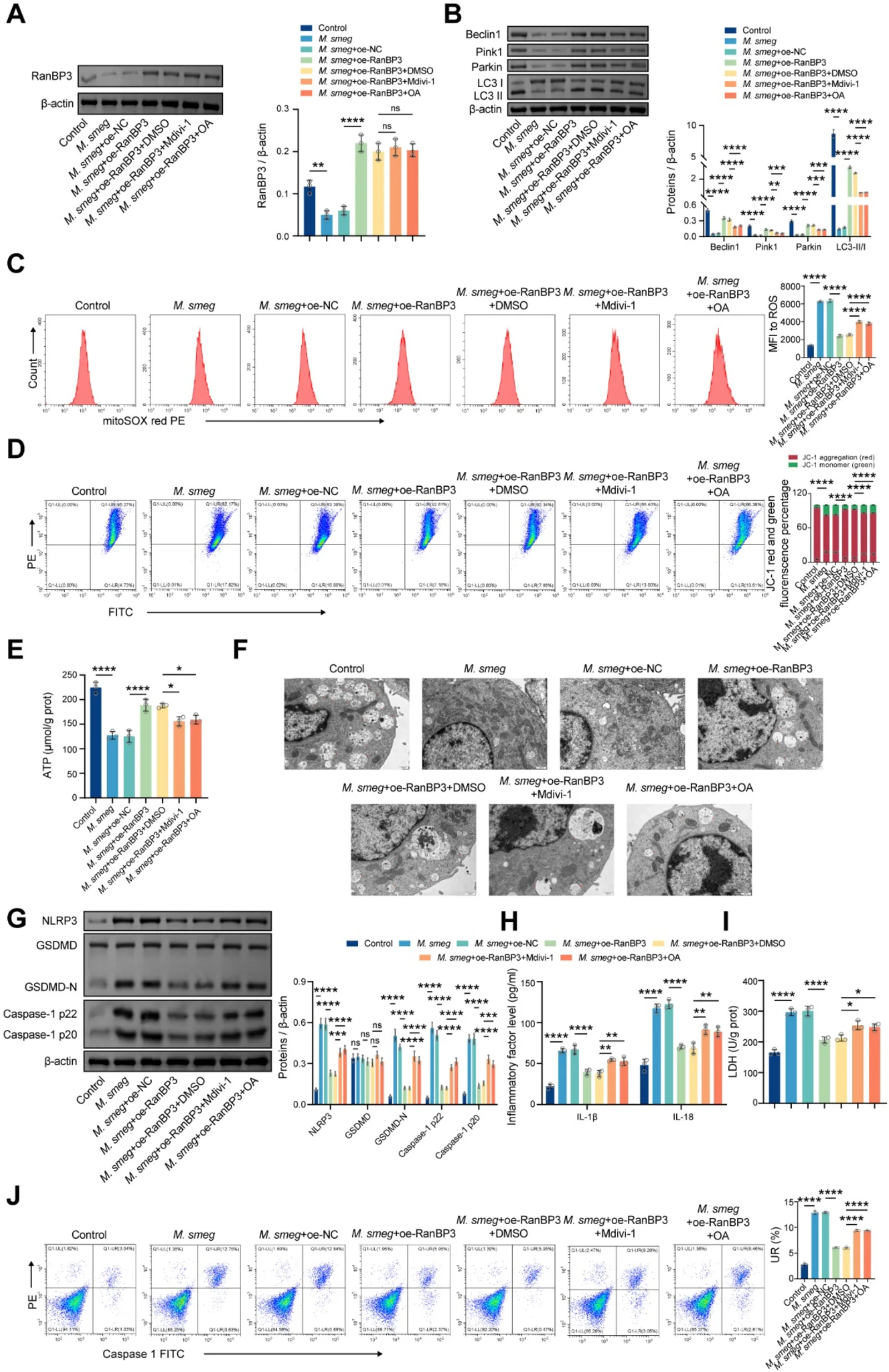
Overexpression of RanBP3 alleviates macrophage pyroptosis and is associated with enhanced mitophagy. BMDMs were transfected with overexpression plasmids for 24 h, then pretreated with Mdivi-1 or OA for 24 h, followed by infection with *M. smegmatis* (MOI = 2) for 4 h. Based on the above treatments, the following assays were performed. (**A**) WB detection of RanBP3 protein levels in BMDMs. (**B**) WB detection of LC3-II/I, Beclin1, PINK1, and Parkin protein levels in BMDMs. (**C**) Flow cytometry detection of mitochondrial reactive oxygen species levels. (**D**) Flow cytometry detection of mitochondrial membrane potential levels. (**E**) ELISA assay for cellular ATP production levels. (**F**) TEM observation of mitochondrial autophagy levels. The red arrow points to an autophagosome. Scale bar = 500 nm. (**G**) WB detection of GSDMD, GSDMD-N, Caspase-1 p22/p20, and NLRP3 protein levels in BMDMs. (**H**) ELISA detection of IL-1β and IL-18 levels in cell supernatants. (**I**) ELISA detection of LDH levels in cell supernatants. (J) Flow cytometry detection of pyroptosis. *M. smeg*: cells infected with *M. smegmatis*. n = 3. *P < 0.05; **P < 0.01; ***P < 0.001; ****P < 0.0001.

Macrophage pyroptosis frequently occurs in conjunction with mitophagy^20^, which can prevent pyroptosis via a number of different mechanisms^41,42^. To explore whether overexpressing RanBP3 alleviates macrophage pyroptosis by promoting mitophagy, we introduced mitophagy inhibitors Mdivi-1^43^ and Oroxylin A (OA)^44^. The results of WB detection showed that the protein level of RanBP3 was increased in the group transfected with oe-RanBP3, while the transfection efficiency was not affected by the mitophagy inhibitor (Fig. 3A). WB analysis assessed the protein levels of autophagy -related factors Microtubule - associated protein light chain 3-II/I (LC3-II/I), Beclin1, PINK1, and Parkin in cells. In comparison to the Control group, the *M. smeg* group exhibited decreased protein levels of LC3-II/I, Beclin1, PINK1, and Parkin in BMDMs, as indicated by the results. Compared with the *M. smeg* + oe-NC group, overexpression of RanBP3 increased the protein levels of the aforementioned autophagy-related factors in BMDMs, while treatment with Mdivi-1 and OA partially reversed this trend (Fig. 3B). Meanwhile, in comparison to the Control group, the *M. smeg* group upregulated the mitochondrial ROS levels and downregulated the membrane potential levels and ATP levels. When contrasted with the *M. smeg* + oe-NC group, RanBP3 overexpression resulted in decreased mitochondrial ROS levels and increased membrane potential and ATP levels. However, Mdivi-1 and OA treatment alleviated the down-regulation effect of RanBP3 overexpression on mitochondrial ROS levels and the up-regulation effect on membrane potential and ATP levels (Fig. 3C–E). TEM observation results showed that compared to the Control group, the level of mitophagy in the *M. smeg* group was significantly reduced; after overexpressing RanBP3, the level of mitophagy was significantly restored; however, after treatment with Mdivi-1 and OA, the level of mitophagy decreased again (Fig. 3F). WB analysis revealed that the *M.smeg* group exhibited an upregulation of GSDMD-N, Caspase-1 p22/p20, and NLRP3 protein levels compared to the Control group. In contrast, the overexpression of RanBP3 reduced the protein levels of GSDMD-N, Caspase-1 p22/p20, and NLRP3 in comparison to the *M. smeg* + oe-NC group. Interestingly, treatment with Mdivi-1 and OA partially reversed this trend. Any treatment did not change the protein level of GSDMD (Fig. 3G). In addition, compared with the Control group, the *M. smeg* group exhibited increased release levels of IL-1β, IL-18, and LDH. Conversely, RanBP3 overexpression reduced the expression of IL-1β, IL-18, and LDH when compared to the *M. smeg* + oe-NC group. Mdivi-1 and OA treatment partially reversed the down-regulation of IL-1β, IL-18, and LDH release levels caused by RanBP3 overexpression (Fig. 3H and I). In contrast to the Control group, flow cytometry analysis indicated that the proportion of pyroptotic cells was elevated in the *M.smeg* group. In comparison to the *M. smeg* + oe-NC group, RanBP3 overexpression reduced pyroptotic cell proportion, but this effect was partially reversed by Mdivi-1 and OA treatments (Fig. 3J). These findings suggest that RanBP3 overexpression may mitigate macrophage pyroptosis by enhancing mitophagy.

### RanBP3 competitively binds to CCAR2 and disrupts the CCAR2-SIRT1 interaction

**Fig. 4:**
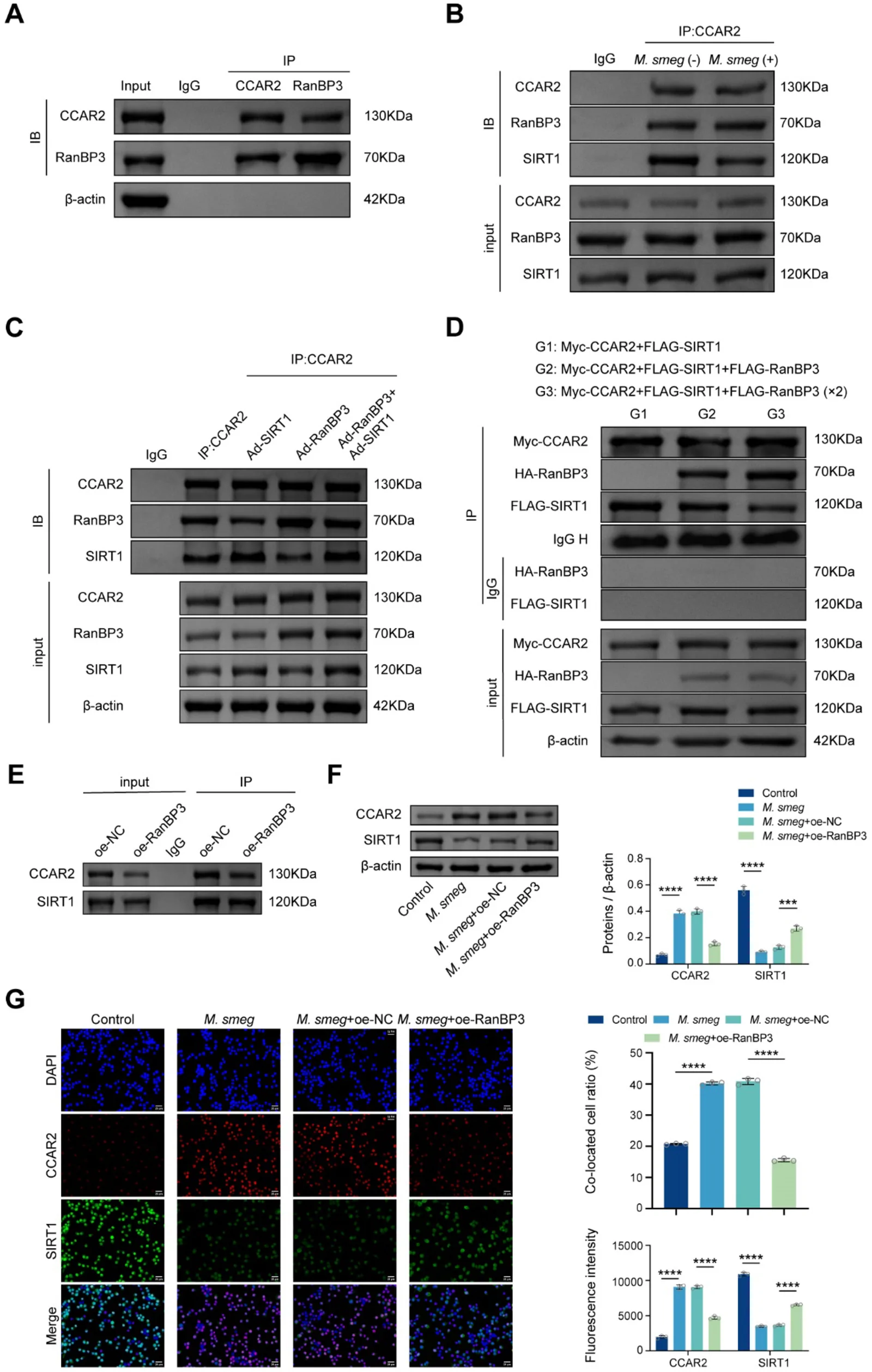
RanBP3 competitively binds to CCAR2 and disrupts the CCAR2-SIRT1 interaction. BMDMs were transfected with overexpression plasmids for 24 h, or transduced with adenovirus for 24 h, where indicated, followed by infection with *M. smegmatis* (MOI = 2) for 4 h. Based on the above treatments, the following assays were performed. (**A**) Co-IP validation of RanBP3 and CCAR2 interaction. (**B**) Co-IP detection of interactions between CCAR2, RanBP3, and SIRT1. (**C**) Co-IP analysis of RanBP3, CCAR2, and SIRT1 interactions in BMDMs transfected with adenovirus (Ad)-RanBP3 or Ad-SIRT1. (**D**) Co-IP analysis of RanBP3 competitive binding to CCAR2. (**E**) Co-IP detection of CCAR2 and SIRT1 binding. (**F**) WB detection of CCAR2 and SIRT1 protein levels in BMDMs. (G) Immunofluorescence detection of localization of CCAR2 and SIRT1 in BMDMs. Scale bar = 25 μm. *M. smeg*: cells infected with *M. smegmatis*. *n* = 3. ***P < 0.001; ****P < 0.0001.

Studies have shown that CCAR2 inhibits SIRT1 activity, and blocking its interaction can activate SIRT1 and autophagy^28^. Upregulating SIRT1 can inhibit pyroptosis by mediating the NLRP3/caspase-1 pathway, thereby protecting cells and tissues^45^. Co-IP results showed that RanBP3 can bind to CCAR2 in BMDMs (Fig. 4A and Figure S2A). The amount of endogenous RanBP3-CCAR2 complexes in *M. smeg*-stimulated BMDMs was significantly increased, while CCAR2-SIRT1 protein levels significantly decreased (Fig. 4B and Figure S2B). Therefore, we speculated that CCAR2 serves as a common binding partner for RanBP3 and SIRT1, and RanBP3 likely binds to CCAR2 in a competitive manner to disrupt the interaction between CCAR2-SIRT1. We transfected BMDMs with oe-RanBP3 adenovirus (Ad-RanBP3) or oe-SIRT1 adenovirus (Ad-SIRT1), and the results showed that RanBP3 overexpression disrupted the CCAR2-SIRT1 interaction, whereas SIRT1 overexpression similarly reduced the CCAR2-RanBP3 association. Importantly, when RanBP3 and SIRT1 were co-overexpressed, RanBP3 binding to CCAR2 significantly decreased while SIRT1 binding increased compared to RanBP3 overexpression alone. These reciprocal changes support the competitive combination of RanBP3 and SIRT1 with CCAR2 (Fig. 4C and Figure S2C). Overexpression of RanBP3 in a dose-dependent manner in the competitive Co-IP experiment showed that the interaction between CCAR2 and SIRT1 was reduced, which further confirmed that RanBP3 competed with SIRT1 for binding to CCAR2. (Fig. 4D and Figure S2D). Overexpression of RanBP3 also reduced the protein levels of the CCAR2-SIRT1 complex (Fig. 4E and Figure S2E). WB detection results showed that after *M. smeg* stimulation, CCAR2 protein level decreased and SIRT1 protein level increased in RanBP3-overexpressing BMDMs (Fig. 4F). Immunofluorescence detection results showed that compared with the Control group, the fluorescence intensity of CCAR2 in the *M. smeg* group increased, while the fluorescence intensity of SIRT1 decreased. However, the overexpression of RanBP3 reversed this trend, resulting in the decrease of CCAR2 intensity and the increase of SIRT1 intensity. In addition, after *M. smegmatis* infection, the proportion of co-located cells of CCAR2 and SIRT1 increased significantly, while the overexpression of RanBP3 significantly decreased this proportion in BMDMs (Fig. 4G). In summary, the above results indicate that RanBP3 inhibits CCAR2 expression by competitively binding CCAR2 with SIRT1.

### The CCAR2/SIRT1 pathway is involved in the pyroptosis of macrophages infected with *M. smegmatis*

**Fig. 5:**
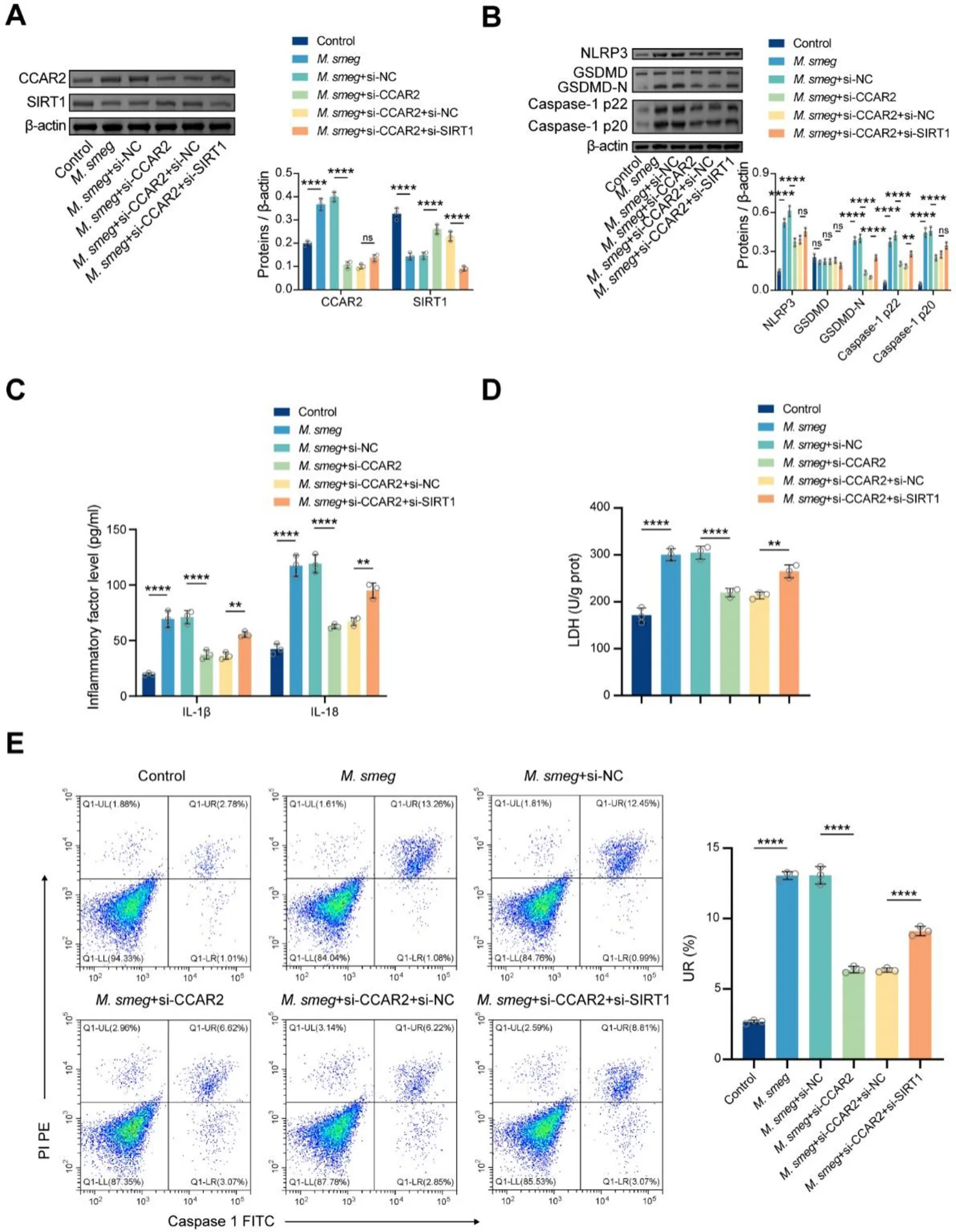
The CCAR2/SIRT1 pathway is involved in pyroptosis of macrophages infected with *M. smegmatis*. BMDMs were transfected with a CCAR2 silencing plasmid, a SIRT1 silencing plasmid, their combination, or negative control siRNA for 24 h as indicated, followed by infection with *M. smegmatis* (MOI = 2) for 4 h. Based on the above treatments, the following assays were performed. (**A**) WB detected the protein levels of CCAR2 and SIRT1 in BMDMs. (**B**) WB detected the protein levels of GSDMD, GSDMD-N, Caspase-1 p22/p20, and NLRP3 in BMDMs. (**C**) ELISA was employed to measure the concentrations of IL-1β and IL-18 in the cellular supernatant. (**D**) ELISA was used to detect the level of LDH in the cell supernatant. (**E**) Flow cytometry was used to detect pyroptosis. *M. smeg*: cells infected with *M. smegmatis*. n = 3. *P < 0.05; **P < 0.01; ****P < 0.0001.

To explore whether the CCAR2/SIRT1 pathway is involved in macrophage pyroptosis infected with *M. smegmatis*, we detected the changes in macrophage pyroptosis after CCAR2 or SIRT1 inhibition (by transfecting with si-CCAR2 or si-SIRT1). qRT-PCR and WB verification showed that compared with the si-NC group, the specific siRNA significantly reduced the mRNA and protein levels of CCAR2 and SIRT1, and the siRNA with the highest knockdown efficiency was selected for subsequent experiments (Figure S1C, D). In addition, after si-CCAR2 treatment, the protein level of CCAR2 was down-regulated. On the other hand, SIRT1 protein level was downregulated with si-SIRT1 administration, which confirmed that si-CCAR2 and si-SIRT1 were effectively knocked down in cells (Fig. 5A). Compared to the Control group, the *M. smeg* group showed an upregulation in the protein levels of pyroptosis-related markers GSDMD-N, Caspase-1 p22/p20, and NLRP3. When compared to the *M. smeg* + si-NC group, knockdown of CCAR2 led to a reduction in the protein levels of GSDMD-N, Caspase-1 p22/p20, and NLRP3. However, the effect of CCAR2 knockdown on the protein levels of these pyroptosis-related factors was partially mitigated by si-SIRT1 treatment. Importantly, there were no notable variances in GSDMD levels across the different groups (Fig. 5B). ELISA results showed that compared with the Control group, the *M. smeg* group upregulated the release levels of IL-1β, IL-18, and LDH. Compared with the *M. smeg* + si-NC group, CCAR2 knockdown downregulated the release levels of IL-1β, IL-18, and LDH. However, after si-SIRT1 treatment, the downregulation effect of CCAR2 knockdown on IL-1β, IL-18, and LDH release levels was attenuated (Fig. 5C and D). Additionally, flow cytometry results showed that the proportion of pyroptotic cells increased in the *M.smeg* group compared to the Control group. Compared to the *M. smeg* + si-NC group, CCAR2 knockdown reduced the proportion of pyroptotic cells. However, this trend was partially reversed after si-SIRT1 treatment (Fig. 5E). These findings show that the CCAR2/SIRT1 pathway is a key regulator of *M. smegmatis*-infected macrophage pyroptosis.

### RanBP3-mediated activation of SIRT1 is associated with enhanced mitophagy

**Fig. 6:**
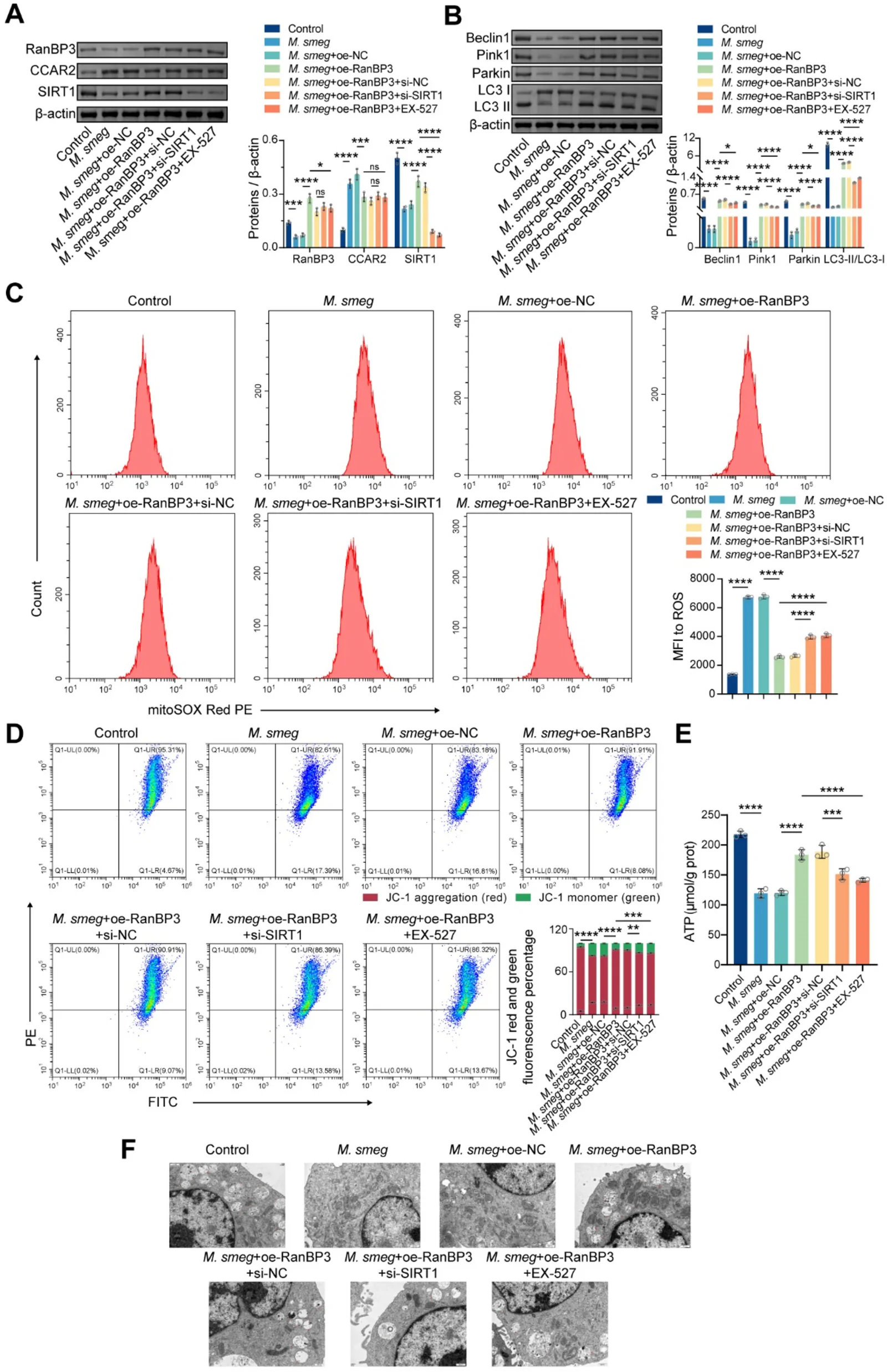
RanBP3-mediated activation of SIRT1 is associated with enhanced mitophagy. BMDMs were co-transfected with an overexpression plasmid and/or a silencing plasmid as indicated, then treated with EX-527 for 1 h, followed by infection with *M. smegmatis* (MOI = 2) for 4 h. Based on the above treatments, the following assays were performed. (**A**) WB detection of RanBP3, CCAR2, and SIRT1 protein levels in BMDMs. (**B**) WB detection of LC3-II/I, Beclin1, PINK1, and Parkin protein levels in BMDMs. (**C**) Flow cytometry detection of mitochondrial reactive oxygen species levels. (**D**) Flow cytometry detection of mitochondrial membrane potential levels. (**E**) ELISA assay for cell ATP production levels. (**F**) TEM observation of mitochondrial autophagy levels. The red arrow points to an autophagosome. Scale bar = 500 nm. *M. smeg*: cells infected with *M. smegmatis*. n = 3. *P < 0.05; ***P < 0.001; ****P < 0.0001.

Next, to further examine whether RanBP3 activates mitophagy by releasing SIRT1, we utilized the SIRT1 enzyme activity inhibitor EX-527 and si-SIRT1 in BMDMs. The results showed decreased SIRT1 protein level in the *M. smeg* group compared to the Control group. In contrast, compared to the *M. smeg* + oe-NC group, RanBP3 overexpression led to increased SIRT1 protein level in BMDMs, whereas treatment with EX-527 and si-SIRT1 both resulted in reduced SIRT1 protein level in BMDMs (Fig. 6A). Overexpression of RanBP3 up-regulated the down-regulated LC3-II/I, Beclin1, PINK1, and Parkin in the *M. smeg* + oe-NC group, but EX-527 and si-SIRT1 treatment partially reversed the up-regulating effect of RanBP3 overexpression on the aforementioned autophagy proteins (Fig. 6B). Similarly, RanBP3 overexpression reduced mitochondrial ROS levels and increased membrane potential and ATP release levels in the *M. smeg* + oe-NC group. However, the effects of RanBP3 overexpression were partially reversed by EX-527 and SIRT1 knockdown (Fig. 6C–E). TEM observations further showed that, compared to the Control group, the levels of mitophagy in the *M. smeg* group were significantly reduced; RanBP3 overexpression significantly restored the levels of mitophagy, while EX-527 or si-SIRT1 treatment partially reversed this restoration (Fig. 6F). These results provide supportive evidence that RanBP3 promotes mitophagy through activation of SIRT1.

### RanBP3 alleviates TBTB in mice via the CCAR2/SIRT1 axis

**Fig. 7:**
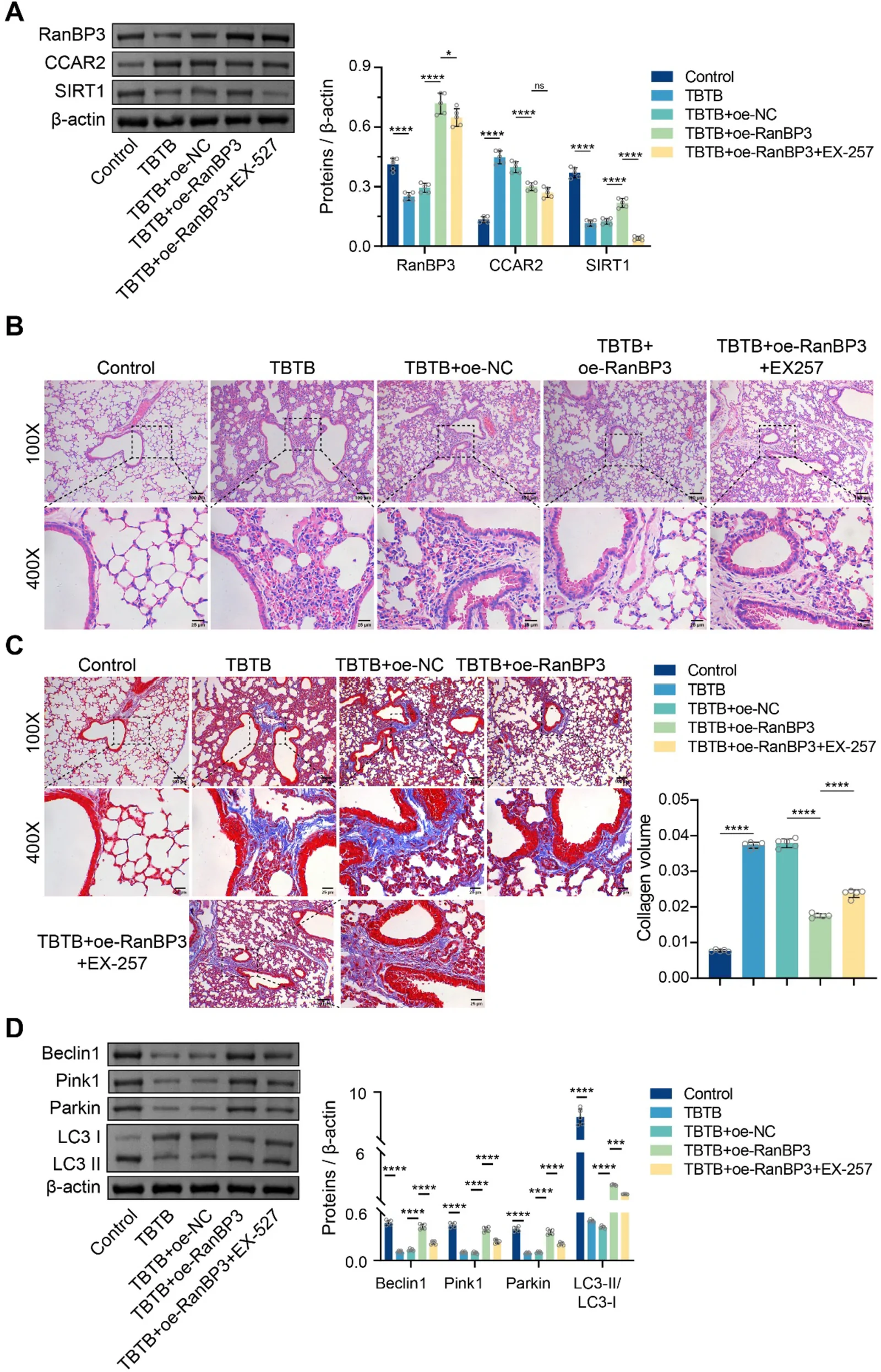

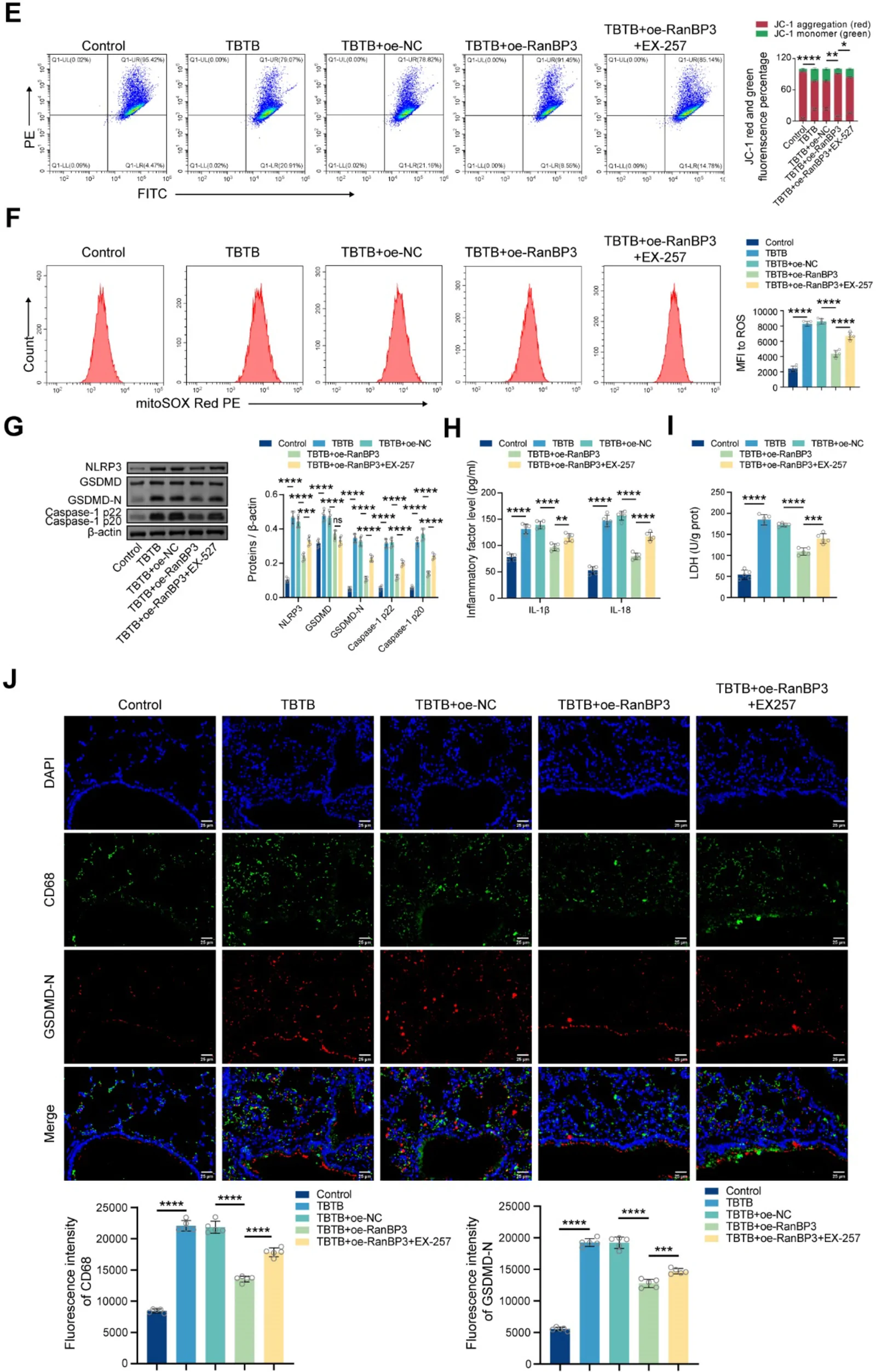
RanBP3 alleviates TBTB in mice via the CCAR2/SIRT1 axis. Mice were intranasally inoculated with 50 µL of *M. smegmatis* suspension (5.4 × 10⁷ ± 0.9 CFU) to establish the TBTB model. Before infection, mice were injected via the tail vein with oe-NC or oe-RanBP3 lentivirus (100 µL, 1 × 10⁸ TU/mL) every two weeks. For SIRT1 inhibition, EX-527 (5 mg/kg) was administered intraperitoneally at the time of infection. After 16 weeks, mice were euthanized, and lung tissues were collected for the following assays. (**A**) WB detection of RanBP3, CCAR2, and SIRT1 protein levels in lung tissue. (**B**) H&E staining assessment of inflammatory damage in lung tissue. (Upper: magnification: ×100, scale bar = 100 μm; Lower: magnification: ×400, scale bar = 25 μm). (**C**) Masson trichrome staining assessment of pulmonary fibrosis. (Upper: magnification: ×100, scale bar = 100 μm; Lower: magnification: ×400, scale bar = 25 μm). (**D**) WB detection of LC3-II/I, Beclin1, PINK1, and Parkin levels in lung tissue. (**E**) Flow cytometry detection of mitochondrial membrane potential levels in tissue sections. (**F**) Flow cytometry detection of reactive oxygen species levels in mitochondrial tissue sections (**G**) WB detection of GSDMD, GSDMD-N, Caspase-1 p22/p20, and NLRP3 protein levels in lung tissue. (**H**) ELISA detection of IL-1β and IL-18 levels in lung tissue homogenate supernatant. (**I**) ELISA detection of LDH levels in lung tissue homogenate supernatant. (**J**) Immunofluorescence CD68 (macrophages) + GSDMD-N (pyroptosis factor) colocalization. Scale bar = 25 μm. n = 5. **P < 0.01; ***P < 0.001; ****P < 0.0001, ns stands for no significant difference.

Finally, we established a TBTB animal model to further validate the effect of RanBP3 overexpression on pyroptosis in TBTB. Consistent with the in vitro experiments, compared with the Control group, RanBP3 and SIRT1 protein levels were downregulated in the TBTB group, while CCAR2 protein level was upregulated. Compared with the TBTB + oe-NC group, RanBP3 overexpression increased SIRT1 protein level and decreased CCAR2 protein level in lung tissue (Fig. 7A). The results of HE and Masson staining showed that the TBTB group had more lung tissue fibrosis and inflammation than the Control group. In comparison to the TBTB + oe-NC group, RanBP3 overexpression mitigated inflammation and fibrosis in the lung tissue of TBTB mice (Fig. 7B and C). Additionally, RanBP3 overexpression resulted in elevated protein levels of LC3-II/I, Beclin1, PINK1, Parkin, and mitochondrial membrane potential in the lung tissue of TBTB mice. However, the effects of RanBP3 overexpression were partially reversed by EX-527 (Fig. 7D and E). Overexpression of RanBP3 decreased the levels of mitochondrial ROS, GSDMD-N, Caspase-1 p22/p20, NLRP3, IL-1β, IL-18, and LDH in the lung tissue of TBTB mice, but EX-527 induction partially counteracted the effects of RanBP3 overexpression. Interestingly, there were no discernible variations in GSDMD protein level between the groups (Fig. 7F–I). Immunofluorescence staining revealed that RanBP3 overexpression significantly reduced the membrane localization of GSDMD-N in CD68 + macrophages in the lung tissue of TBTB mice. Likewise, the effects of RanBP3 overexpression were partially reversed by EX-527 induction (Fig. 7J). These results suggest that RanBP3 may promote mitophagy by competitively binding CCAR2 to release SIRT1 activity, which may contribute to reducing pyroptosis and alleviating TBTB in vivo.

## Discussion

Macrophage pyroptosis can play a dual role in Mtb infection and tuberculosis progression. On the one hand, macrophage pyroptosis may limit Mtb spread by releasing inflammatory factors and recruiting immune cells. However, excessive pyroptosis may lead to tissue damage and chronic inflammation, exacerbating the condition of TBTB^46^. Therefore, how to alleviate excessive macrophage pyroptosis is the key to solving the balance between the two. This study found that the RanBP3 protein level was reduced in the lung mucosal tissue of TBTB patients, and experimental modulation of RanBP3 suggested that it may mitigate macrophage pyroptosis through mitophagy mediated by the CCAR2-SIRT1 axis.

This is the first study to show that RanBP3 protein level is much lower in TBTB patients’ airway mucosal tissue and has a significant negative correlation with pyroptosis markers (IL-1β/IL-18, caspase-1 p22/p20, NLRP3, and GSDMD-N). We discovered that overexpression of RanBP3 lowers LDH levels and the percentage of pyroptotic cells while dramatically inhibiting the protein levels of important pyroptosis indicators in a macrophage model infected with *M. smegmatis*. According to earlier research, mitochondrial dysfunction and disturbed autophagy processes are closely linked to the induction of macrophage pyroptosis^47^. Mitophagy suppresses pyroptosis by clearing damaged mitochondria, reducing ROS production and inflammatory factor release^48^. Mitophagy also suppresses pyroptosis by regulating NLRP3 inflammasome assembly or activity^49^. We conducted an investigation to determine whether the inhibitory effect of RanBP3 on pyroptosis is linked to mitophagy by treating cells with mitophagy inhibitors. Our results indicated that the overexpression of RanBP3 upregulates autophagy-related factors such as LC3-II/I, Beclin1, PINK1, and Parkin, consequently enhancing autophagy activity, reducing mitochondrial ROS accumulation, and maintaining mitochondrial membrane potential and ATP levels. Furthermore, RanBP3 overexpression significantly suppressed the protein levels of pyroptosis-related factors, including GSDMD-N, Caspase-1 p22/p20, NLRP3, IL-1β, IL-18, and LDH. Interestingly, the application of mitophagy inhibitors (Mdivi-1 and oroxylin A) partially reversed the inhibitory effect of RanBP3 on pyroptosis. TEM further showed increased mitophagy levels in RanBP3-overexpressing cells. Prior studies have focused on RanBP3’s role in cell proliferation and tumor suppression^36,39^, but its involvement in pyroptosis and TBTB has not been reported. Our study provided preliminary evidence suggesting that RanBP3 may be involved in regulating the mitophagy-pyroptosis axis via CCAR2-SIRT1.

CCAR2, as a classic inhibitor of SIRT1, plays a regulatory role in diseases such as cancer and metabolic disorders^28^. LNCAROD can inhibit SIRT1 activity by promoting the binding of CCAR2 and AROS, thereby activating mitochondrial apoptosis and exerting its anticancer effects^50^. H_2_S promotes autophagy by inducing the nuclear translocation of GAPDH, thereby disrupting the inhibitory interaction between CCAR2 and SIRT1^28^. IncRNA Snhg9 increases SIRT1 activity by binding to the SIRT1 inhibitory protein CCAR2, thereby inhibiting lipid absorption^51^. According to our research, RanBP3 binds to CCAR2 competitively, breaking the CCAR2-SIRT1 complex and relieving the inhibition of SIRT1 activity. Moreover, during *M. smegmatis* infection, the CCAR2/SIRT1 pathway contributes to macrophage pyroptosis. In addition, overexpression of RanBP3 released SIRT1 by mediating the CCAR2/SIRT1 pathway, up-regulated the protein levels of autophagy-related factors, and down-regulated the protein levels of pyroptosis-related factors, restoring mitochondrial function. However, the consequences of RanBP3 overexpression can be partially reversed by the SIRT1 inhibitor EX-527. Our study suggested that RanBP3 may competitively bind to CCAR2 and activate SIRT1-mediated mitophagy to inhibit macrophage pyroptosis. We first revealed the effect of RanBP3 regulating the CCAR2/SIRT1 axis on cell pyroptosis.

## Limitations

There are also some limitations in this study. Firstly, although the downregulation of RanBP3 in TBTB patient tissues was observed, these data were obtained from whole airway mucosal lysates without distinguishing cell types. Therefore, whether this downregulation specifically occurs within the macrophage population remains unclear, and future cell type-specific validation in clinical samples is needed. Second, although the key role of SIRT1 was confirmed by inhibitor experiments, our evidence for RanBP3-mediated mitophagy is primarily correlative and morphological. Direct measurement of mitophagy flux (e.g., using mt-Keima or mito-QC reporter assays) is required to definitively establish that RanBP3 promotes mitophagy. Moreover, whether RanBP3 synergistically regulates mitophagy through other downstream effector molecules beyond SIRT1 still needs further verification. Third, whether RanBP3 influences pyroptosis via other mechanisms outside the mitophagy pathway still needs to be explored. Finally, it is yet unknown if the CCAR2-SIRT1 axis influences TBTB and is also connected to other cell death pathways (like necroptosis). The above issues will be the focus of our upcoming research.

## Conclusion

In summary, our research suggests that RanBP3 alleviates TBTB by competitively binding CCAR2 to disrupt the CCAR2-SIRT1 complex, which activates SIRT1-mediated mitophagy and inhibits macrophage pyroptosis. This not only expands the functional cognition of RanBP3 in TBTB, but also provides a new therapeutic strategy for the treatment of TBTB with the regulation of mitophagy and pyroptosis as the target.

## Supplementary Information

Below is the link to the electronic supplementary material.

Supplementary Material 1

## Data Availability

The datasets used and analyzed during the current study are available from the corresponding author on reasonable request.
