# Supplementary material for: RANBP3 promotes mitophagy through the CCAR2/SIRT1 pathway to alleviate pyroptosis in macrophages in TBTB
Source: Sci Rep. 2026 Jun 11;16:26707. doi: 10.1038/s41598-026-55696-2 (PMC13507262; doi:10.1038/s41598-026-55696-2)

Fig. 1a

RanBP3 (70KDa) β-actin (42KDa)

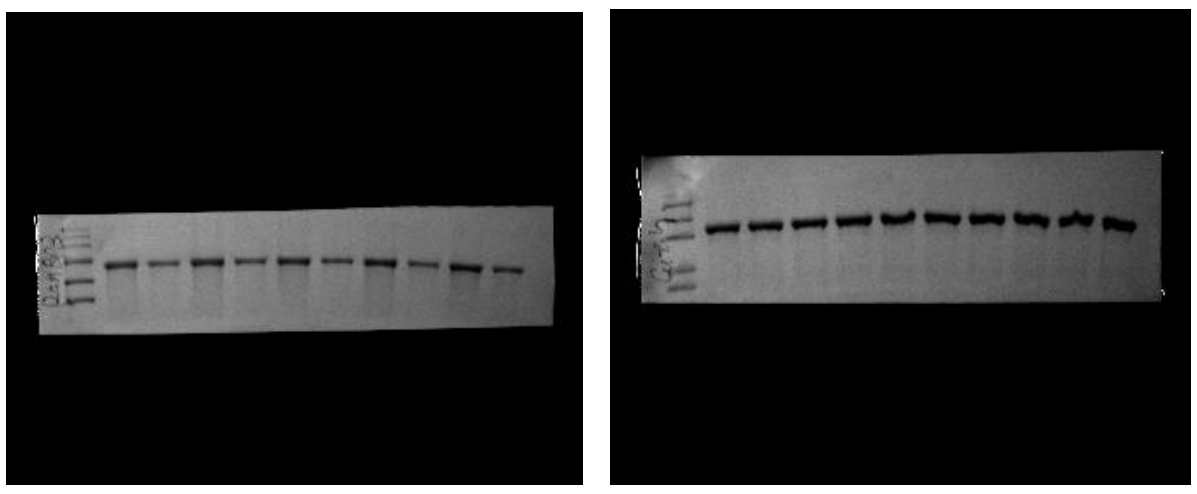

Fig. 1b

NLRP3 (110KDa) GSDMD (47-53,31KDa) Casp1 (22,20KDa) β-actin (42KDa)

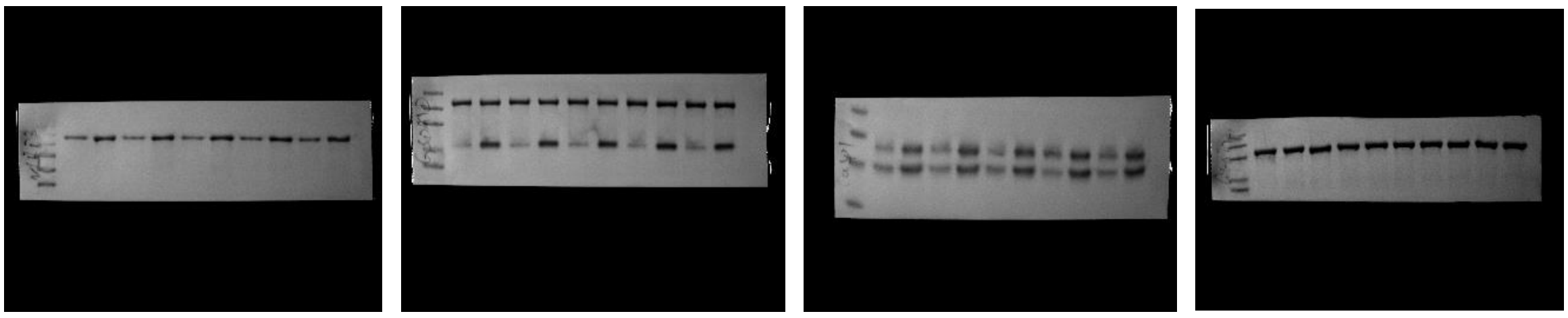

Fig. 2b

RanBP3 (70KDa) β-actin (42KDa)

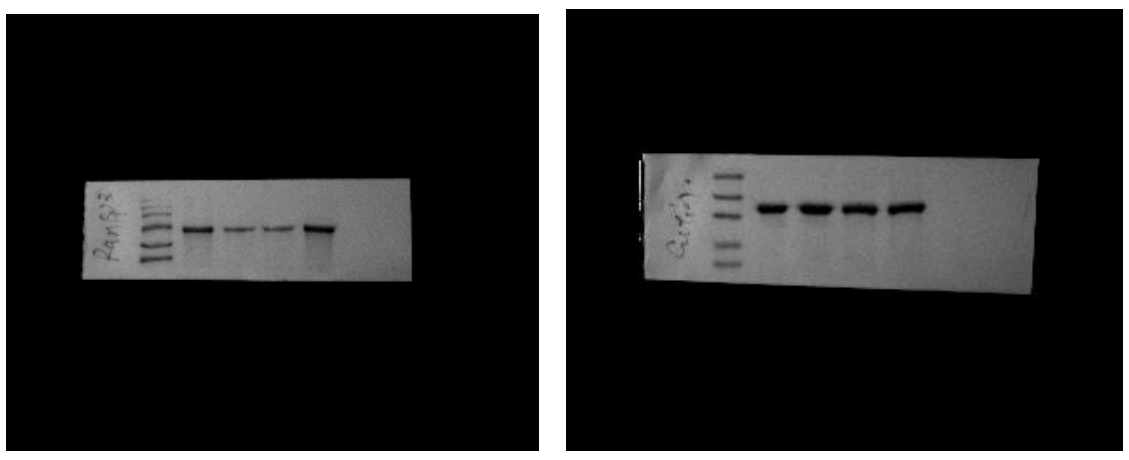

Fig. 2c

NLRP3 (110KDa) GSDMD (47-53,31KDa) Casp1 (22,20KDa) β-actin (42KDa)

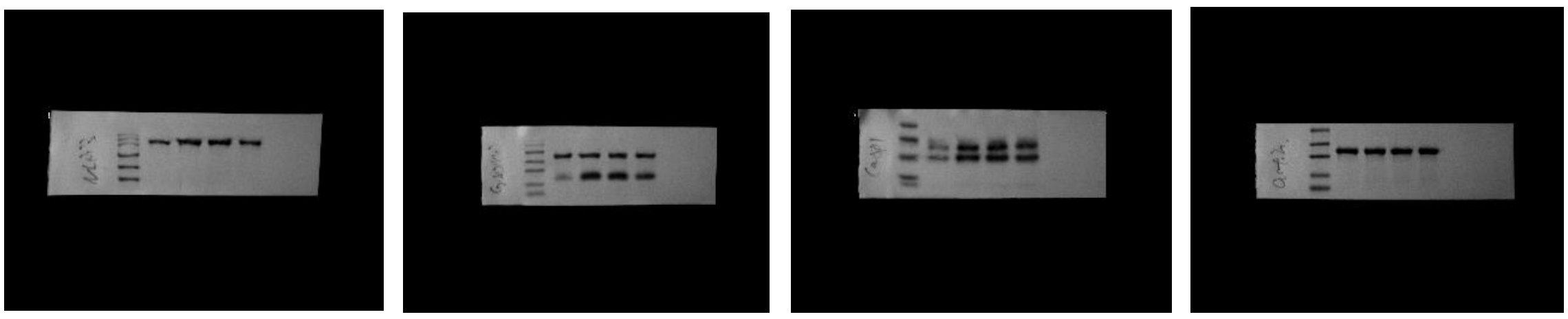

Fig. 3a

RanBP3 (70KDa) β-actin (42KDa)

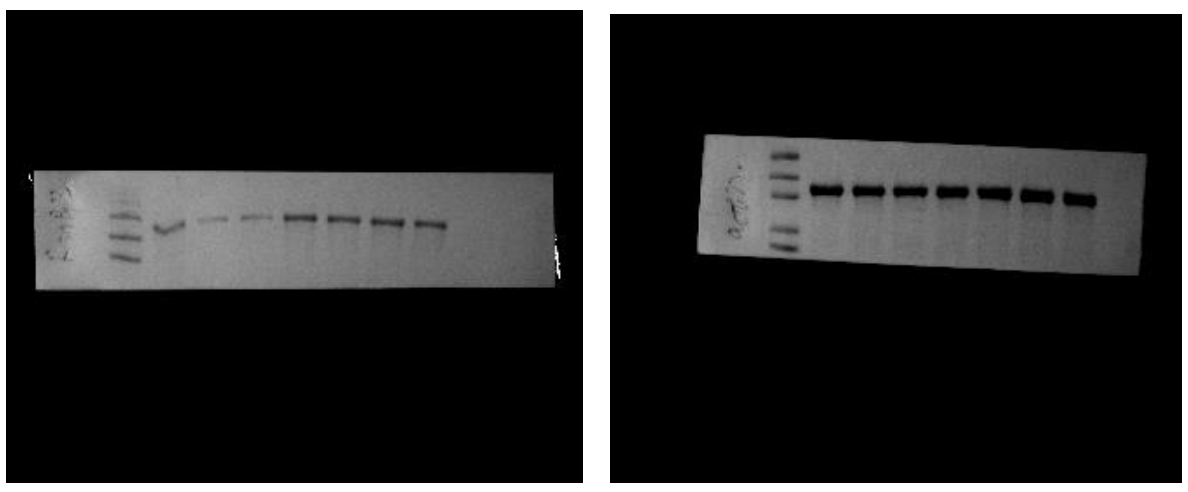

Fig. 3b

Beclin1 (52KDa) Pink1(63KDa) Parkin (55KDa) LC3 (14-18 KDa)

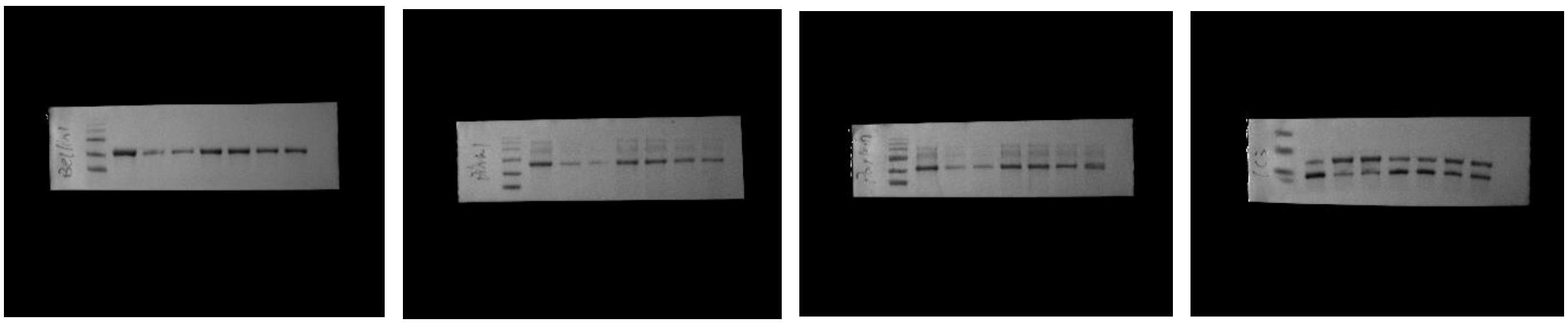

β-actin (42KDa)

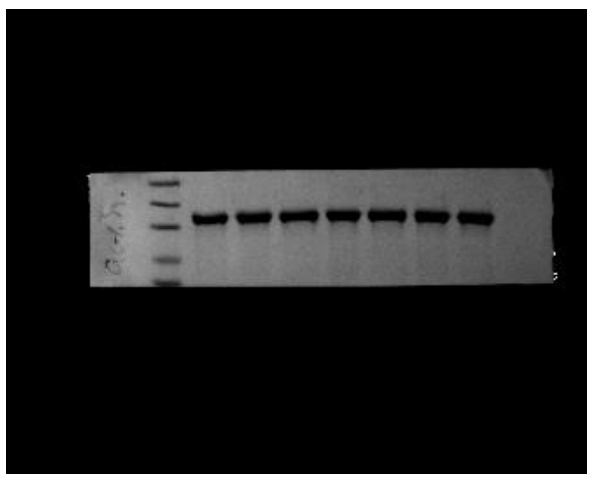

Fig. 3g

NLRP3 (110KDa) GSDMD (47-53,31KDa) Casp1(22,20KDa) β-actin (42KDa)

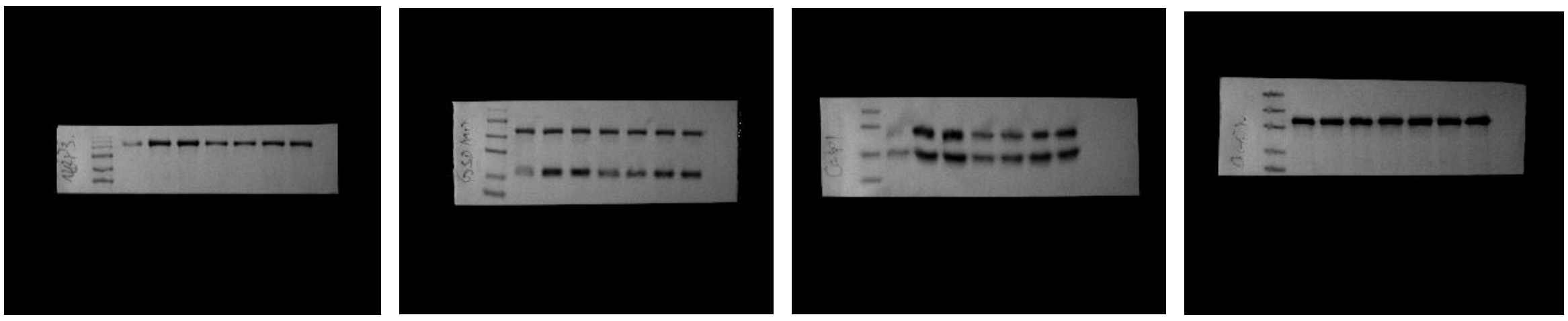

Fig. 4a

CCAR2 (130KDa) RanBP3 (70KDa) β-actin (42KDa)

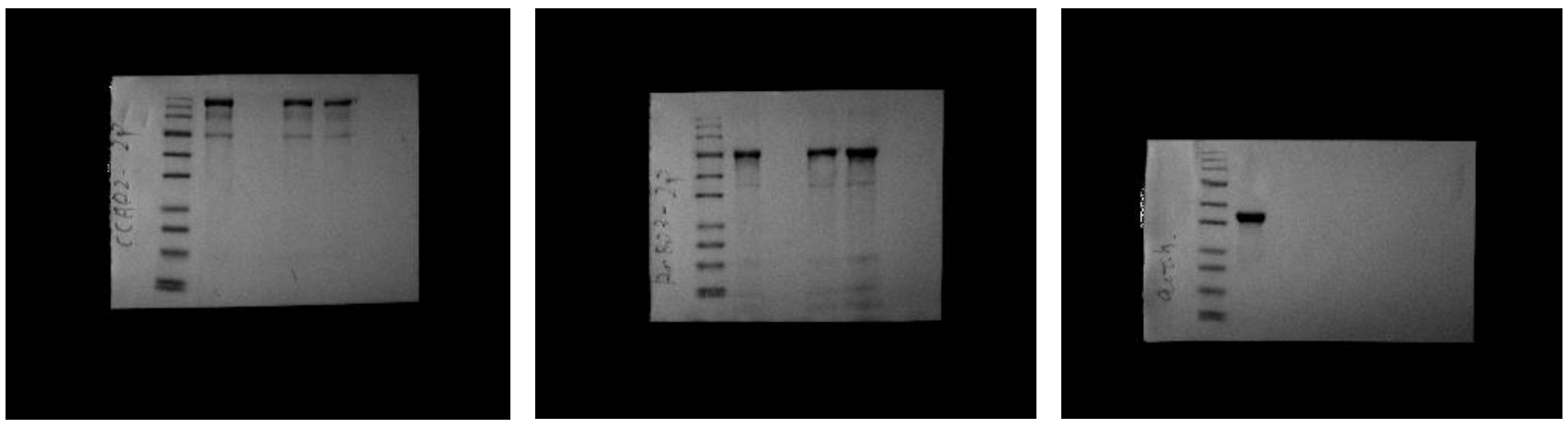

Fig. 4b

IB: CCAR2 (130KDa) RanBP3 (70KDa) SIRT1(120KDa)

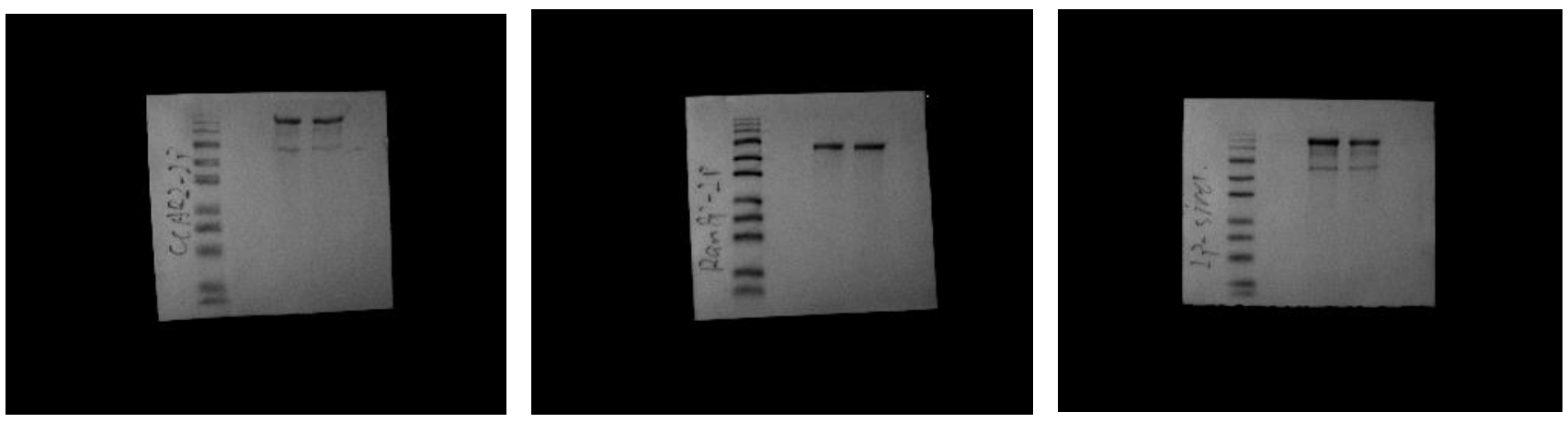

input: CCAR2 (130KDa) RanBP3 (70KDa) SIRT1(120KDa)

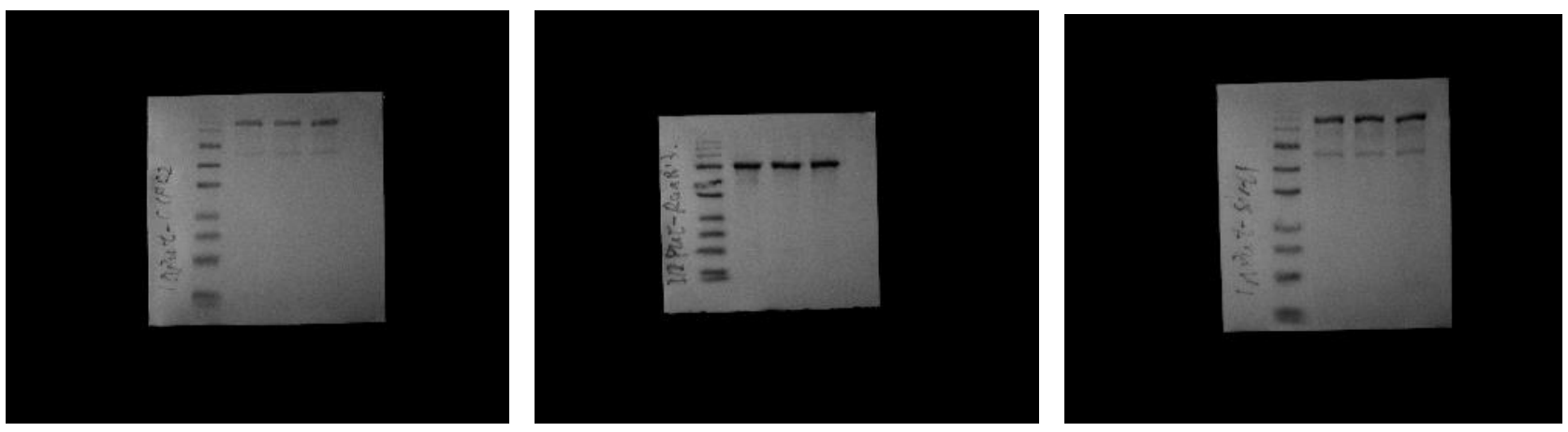

Fig. 4c

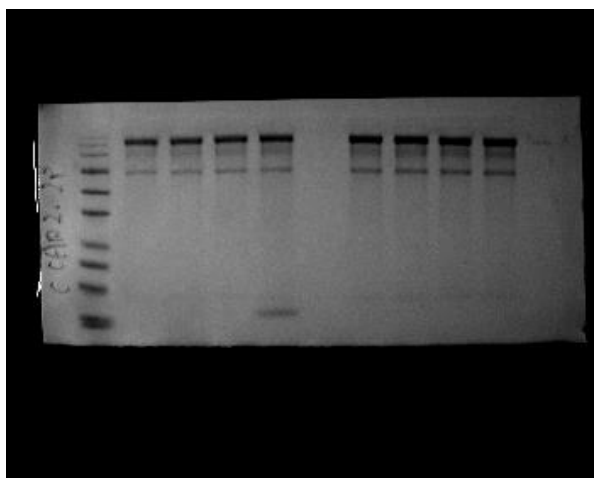
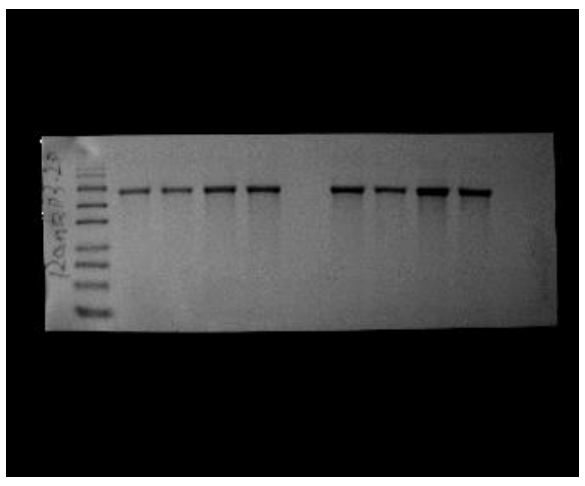
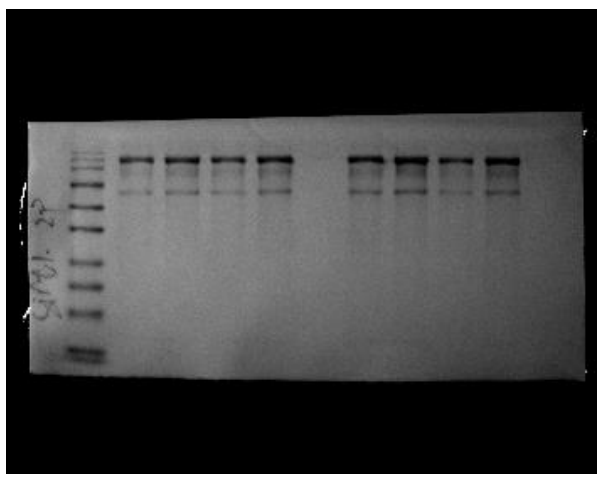
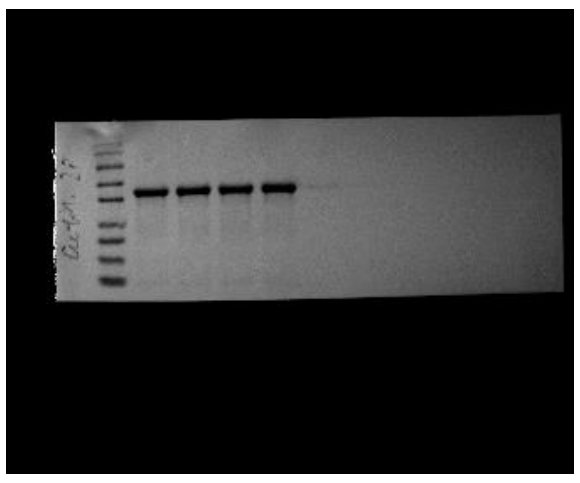

Fig. 4d

IP: myc-CCAR2 (130KDa) HA-RanBP3 (70KDa) FLAG-SIRT1(120KDa) IgG H

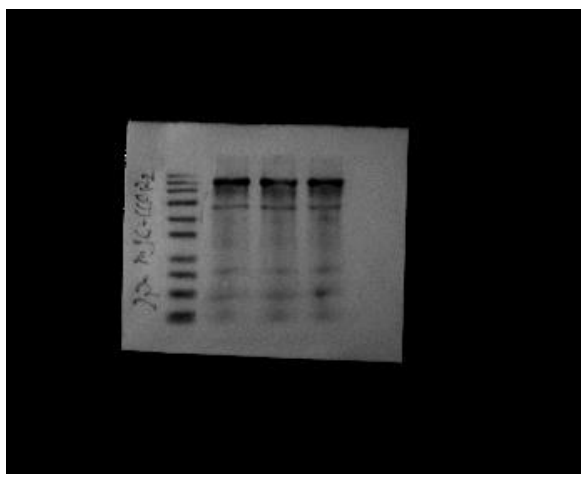
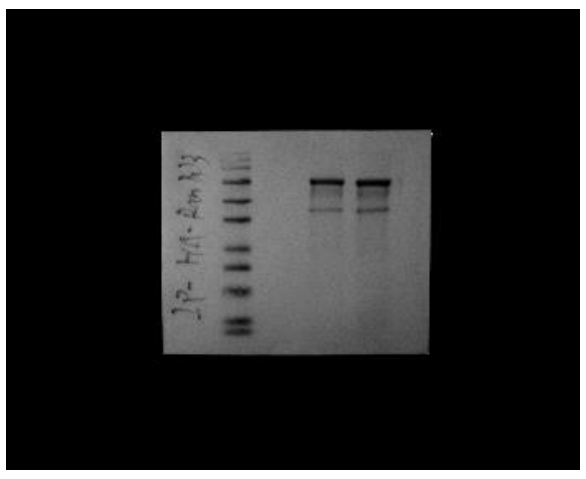
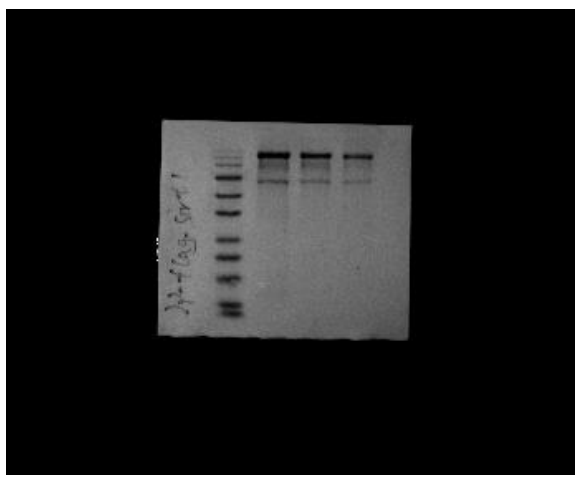
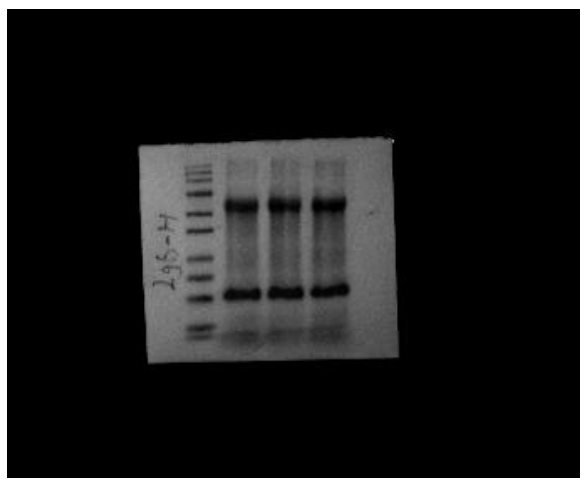

IgG: HA-RanBP3 (70KDa) FLAG-SIRT1(120KDa) input: myc-CCAR2 (130KDa) HA-RanBP3 (70KDa)

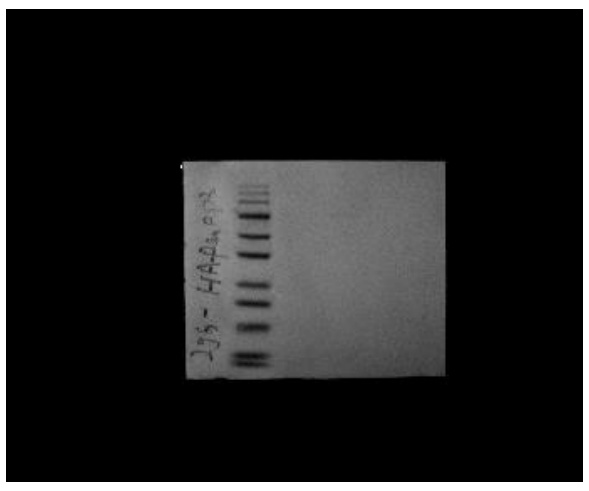
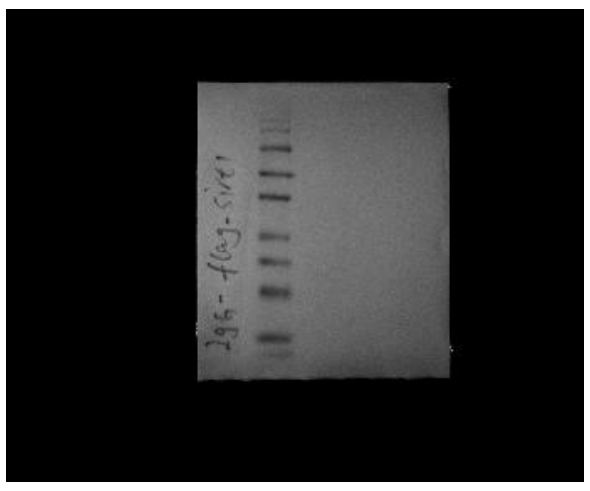
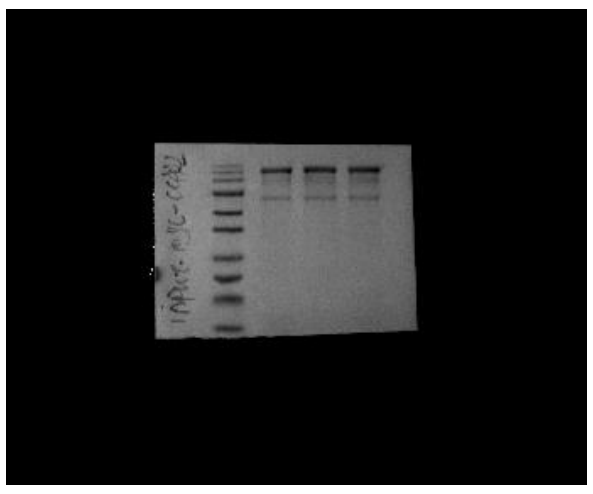
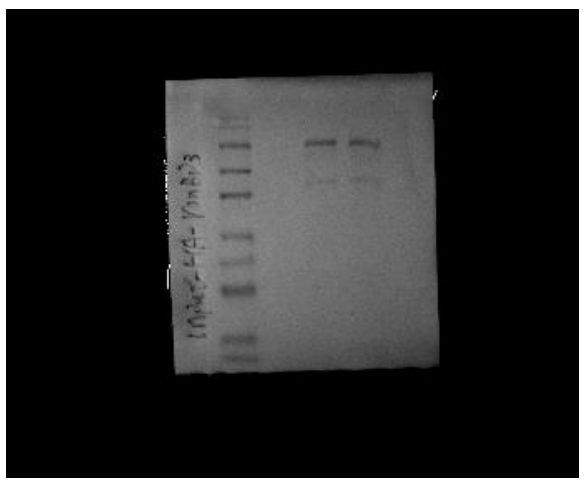

FLAG-SIRT1(120KDa) β-actin (42KDa)

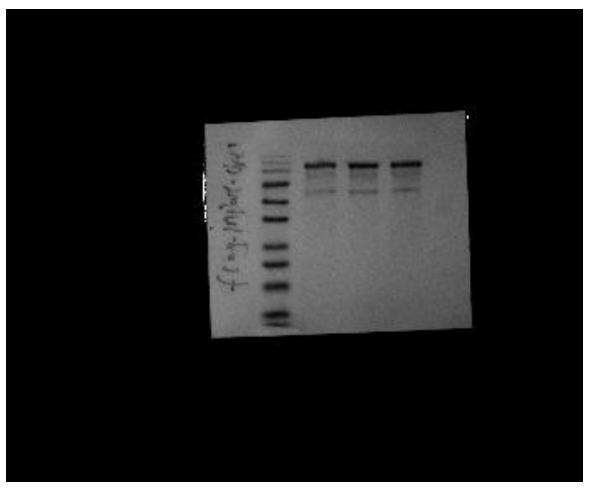
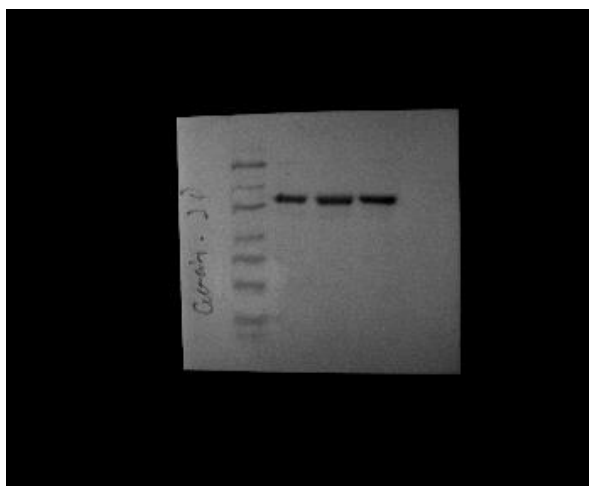

Fig. 4e

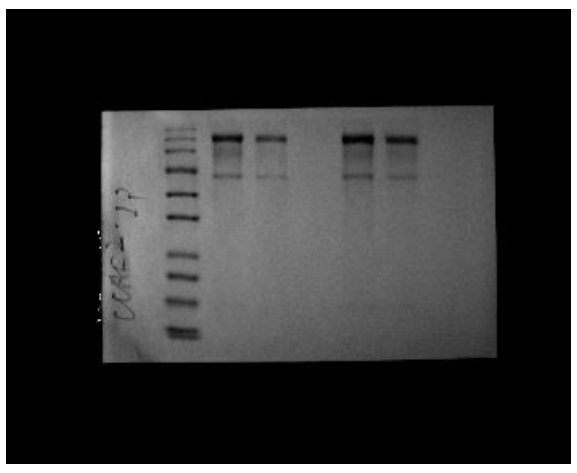
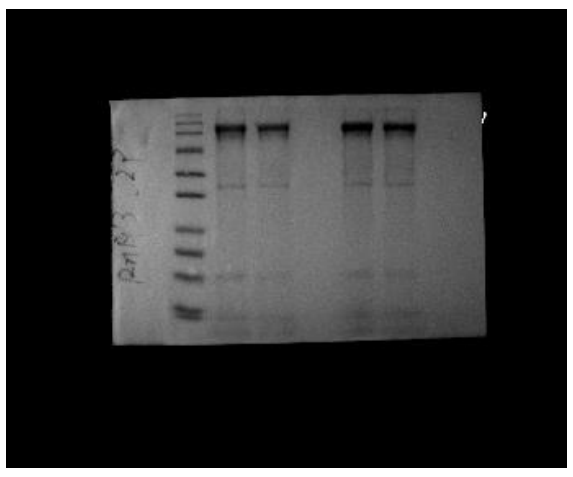

Fig. 4f

CCAR2 (130KDa) SIRT1 (120KDa) β-actin (42KDa)

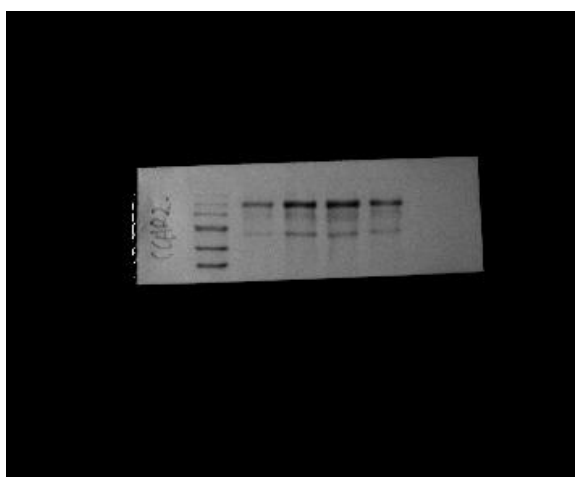
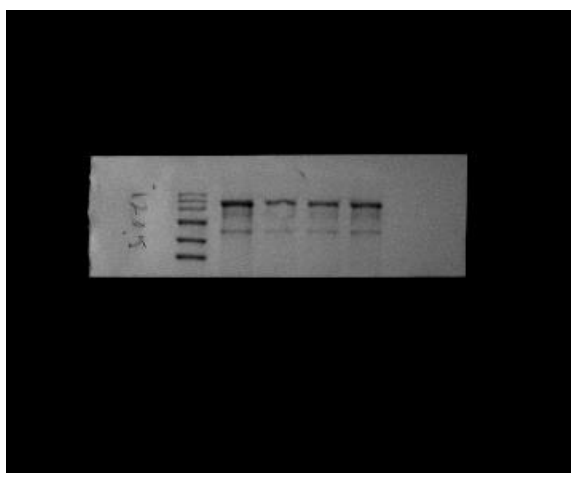
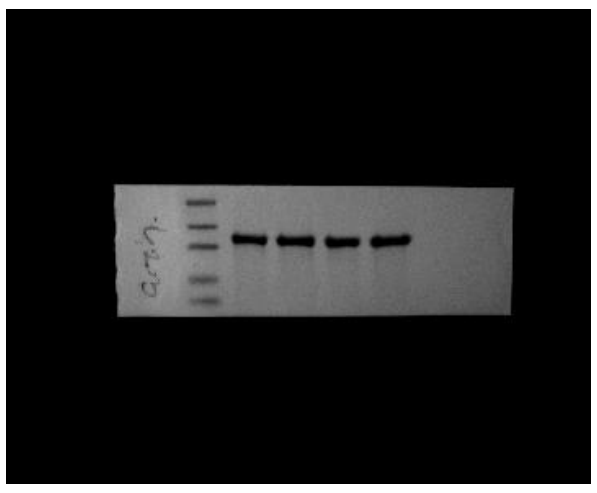

Fig. 5a

CCAR2 (130KDa) SIRT1 (120KDa) β-actin (42KDa)

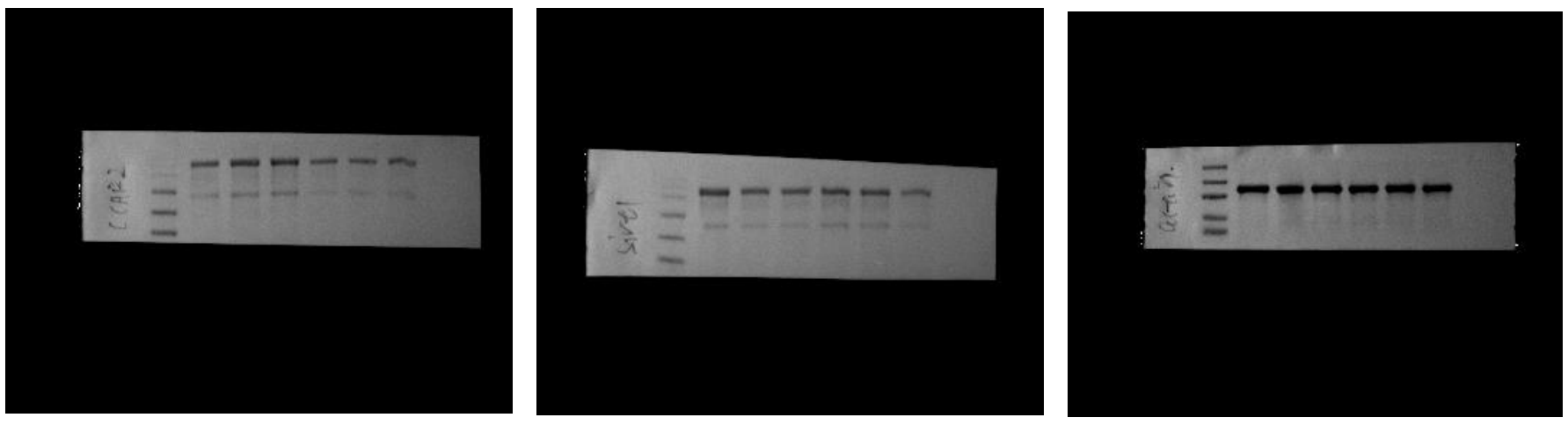

Fig. 5b

NLRP3 (110KDa) GSDMD (47-53,31KDa) Casp1 (22,20KDa) β-actin (42KDa)

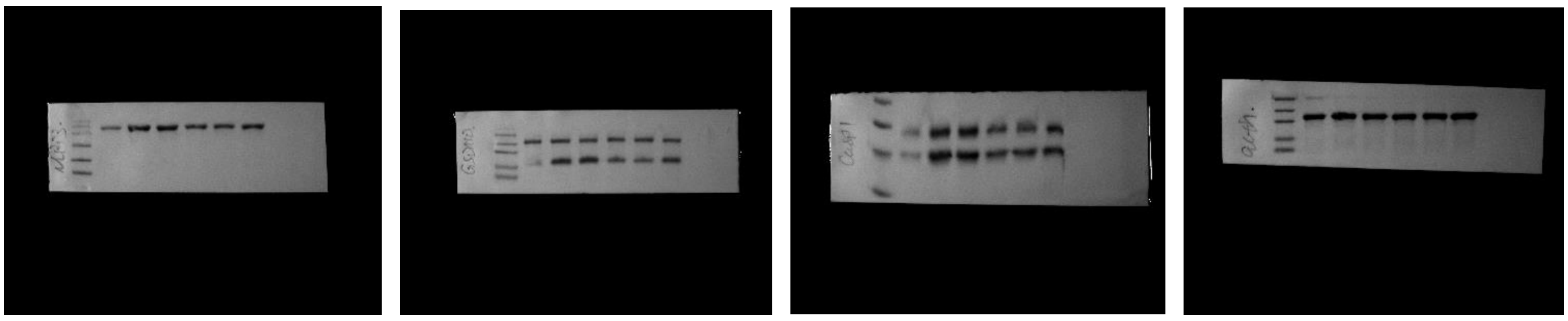

Fig. 6a

RanBP3 (70KDa) CCAR2 (130KDa) SIRT1 (120KDa) β-actin (42KDa)

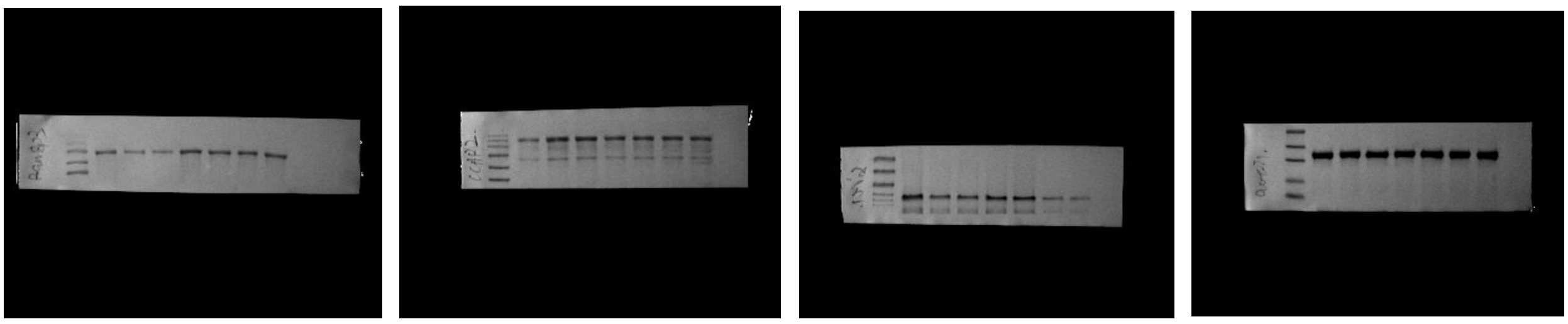

Fig. 6b

Beclin1 (52KDa) Pink1 (63KDa) Parkin (55KDa) LC3 (14-18 KDa)

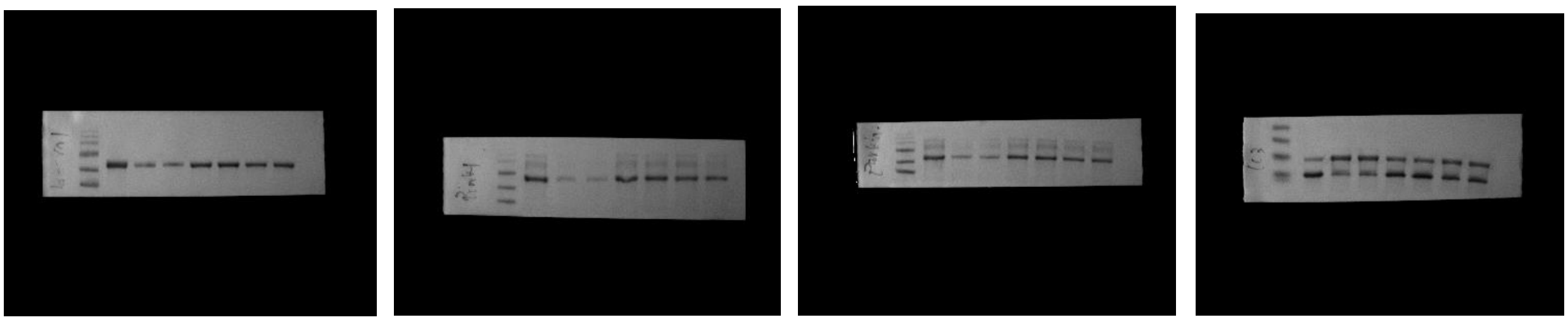

β-actin (42KDa)

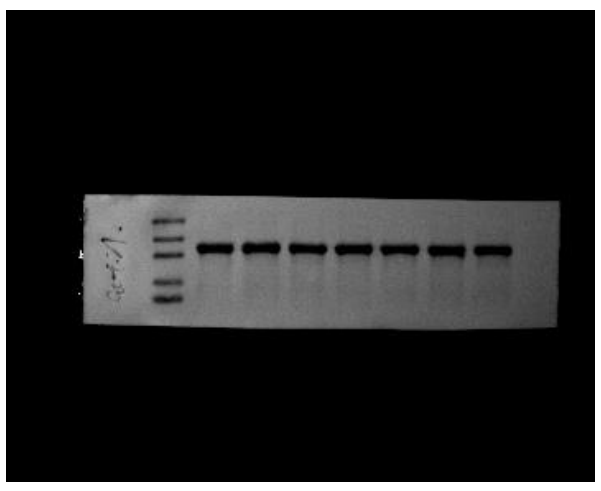

Fig. 7a

RanBP3 (70KDa) CCAR2 (130KDa) SIRT1 (120KDa) β-actin (42KDa)

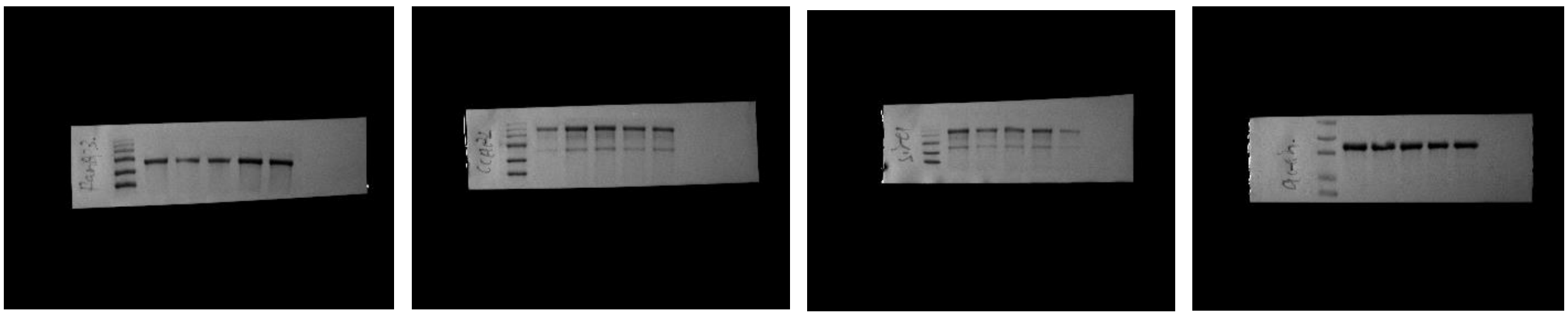

Fig. 7d

Beclin1 (52KDa)

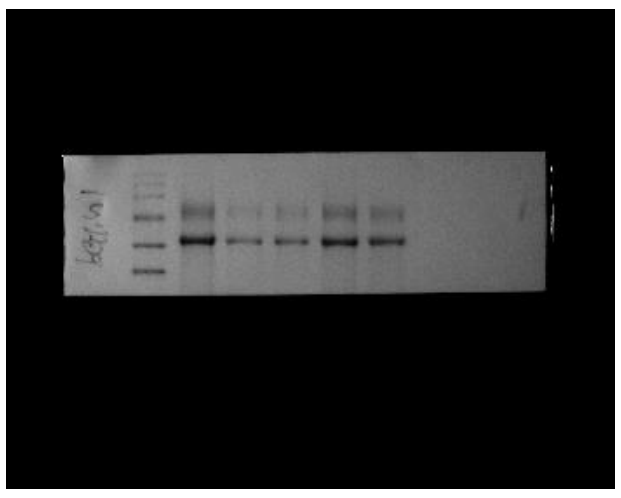

Pink1 (63KDa)

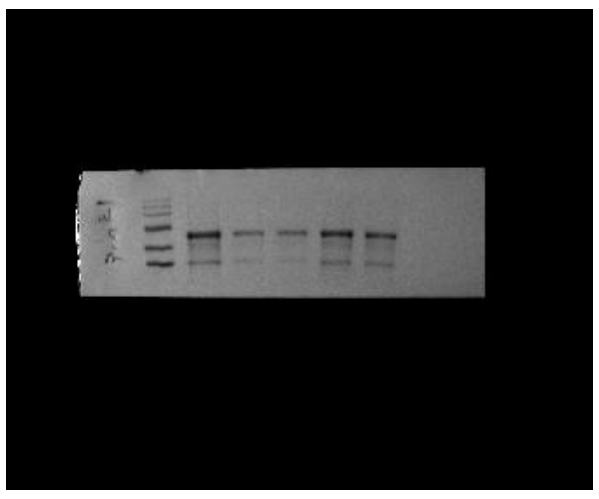

Parkin (55KDa)

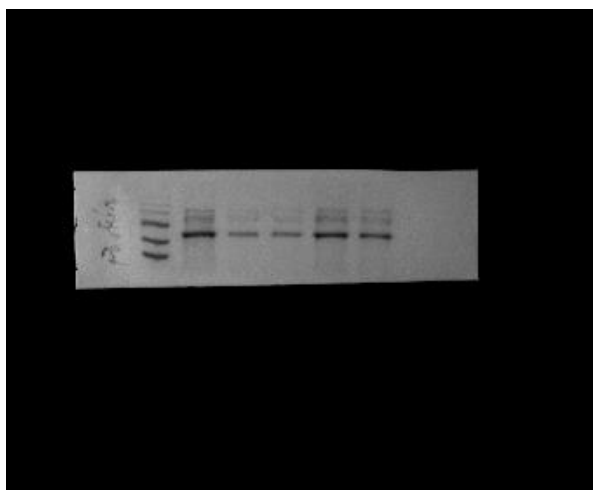

LC3 (14-18 KDa)

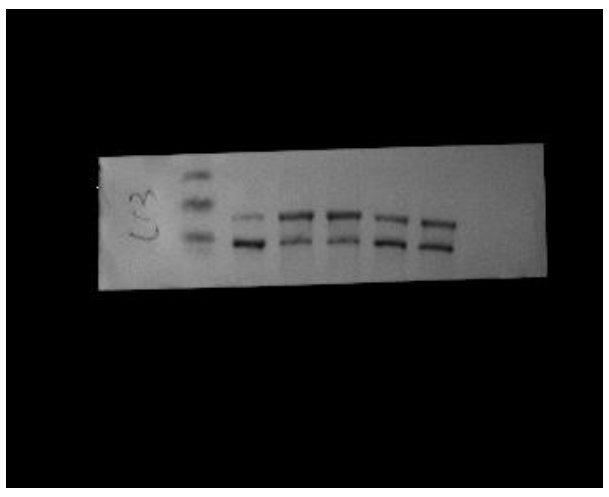

β-actin (42KDa)

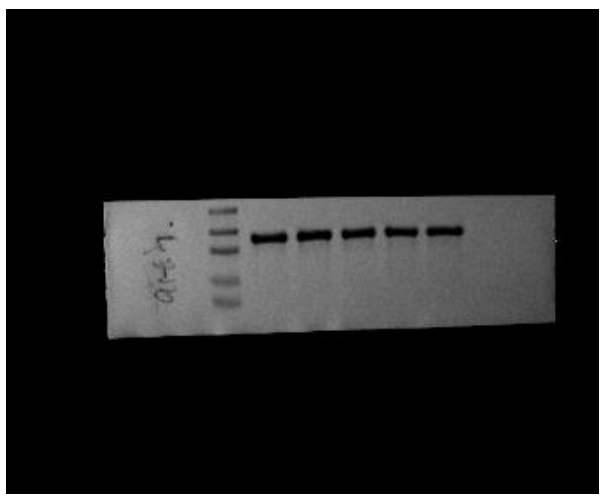

Fig. 7g

NLRP3 (110KDa)

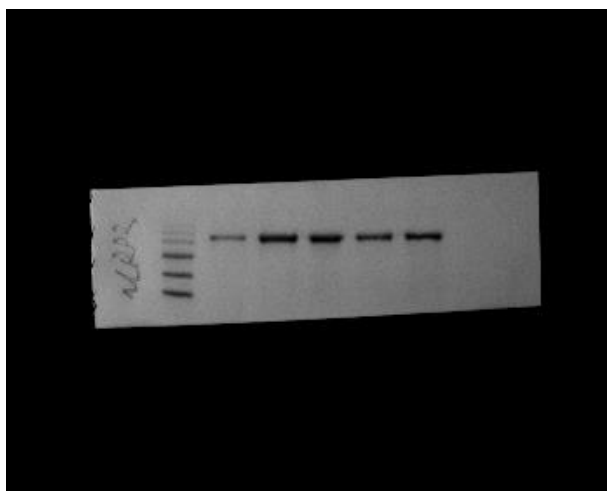

GSDMD (47-53,31KDa)

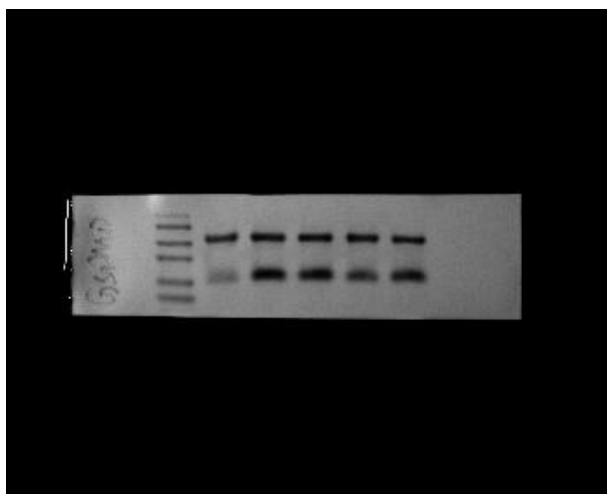

Casp1 (22,20KDa)

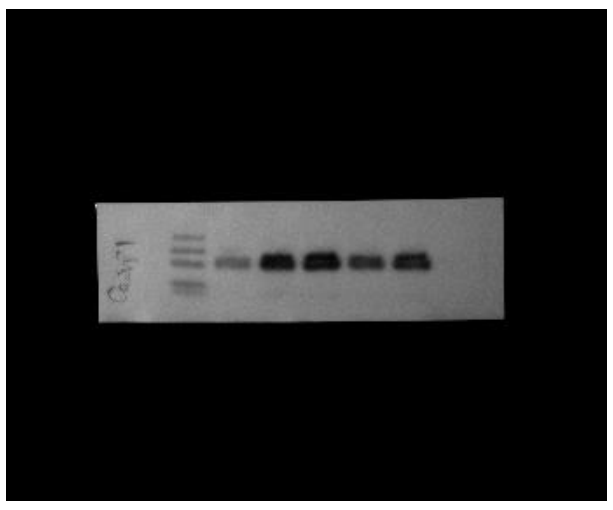

β-actin (42KDa)

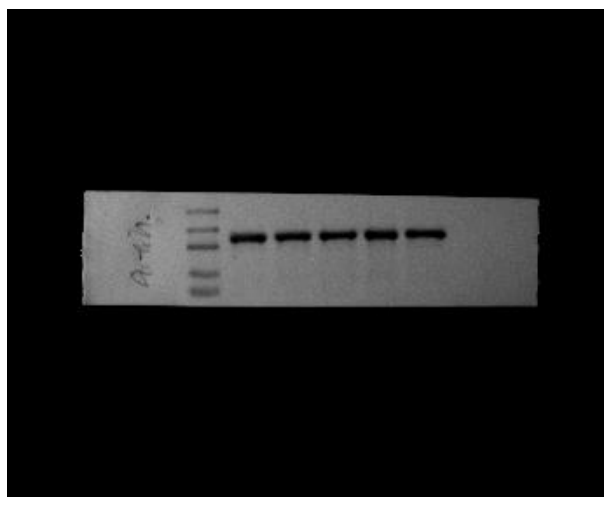

Revised _ All of Original data files of western blots

Fig. 2H

RanBP3 (70KDa) β-actin (42KDa)

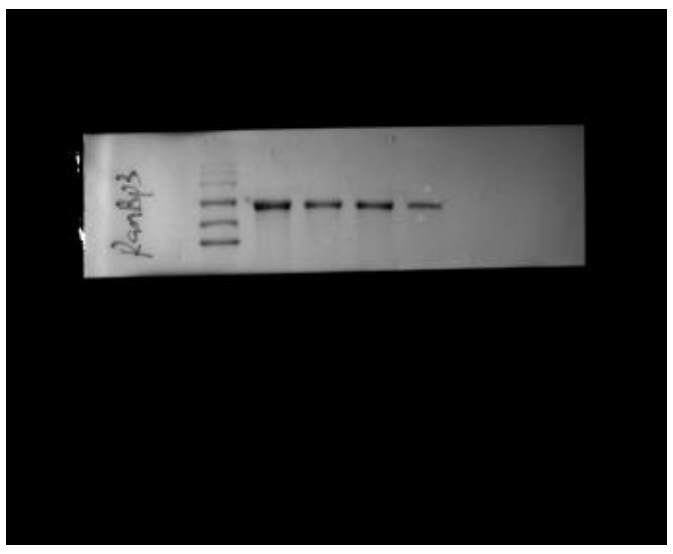

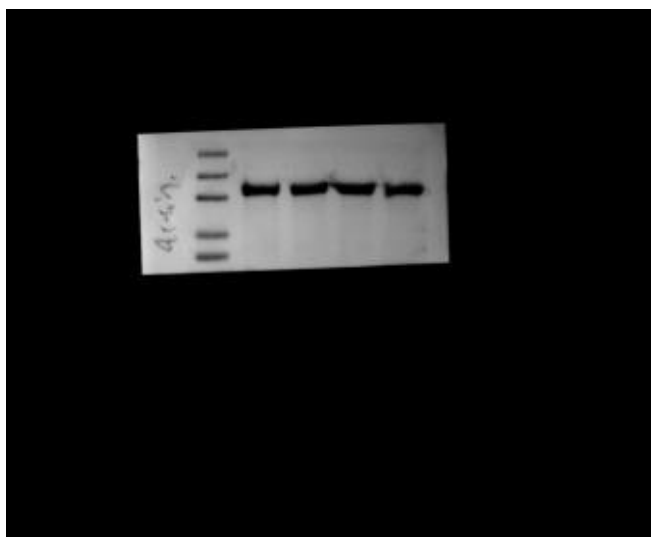

Fig. 2I

NLRP3 (110KDa) GSDMD (47-53,31KDa) Casp1 (22,20KDa) β-actin (42KDa)

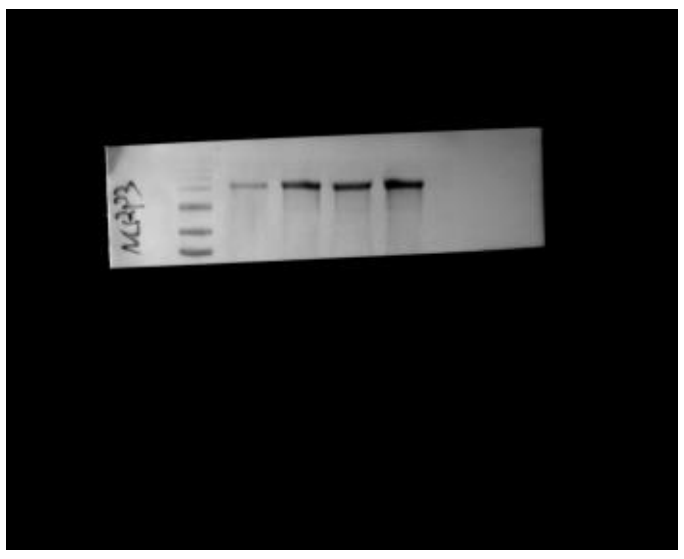

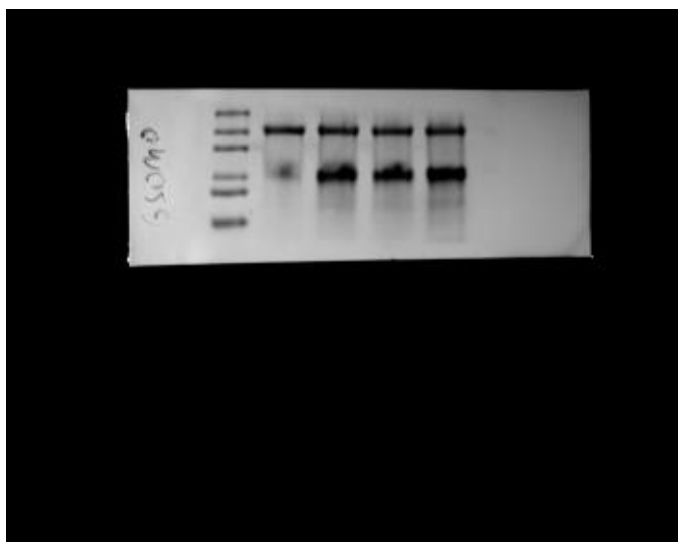

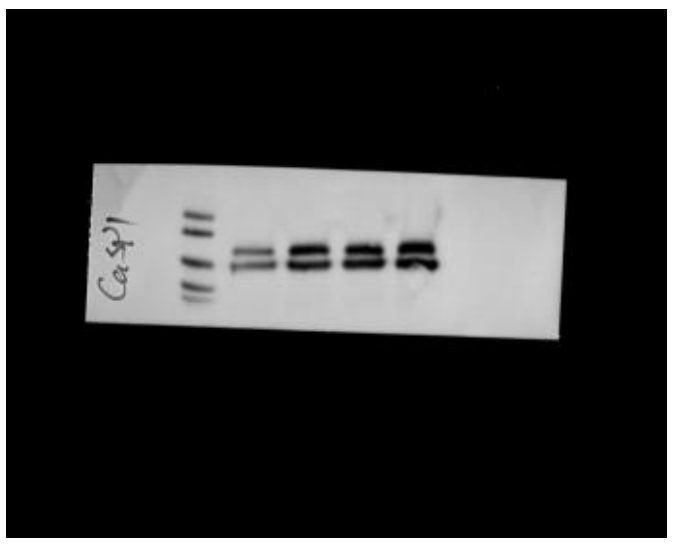

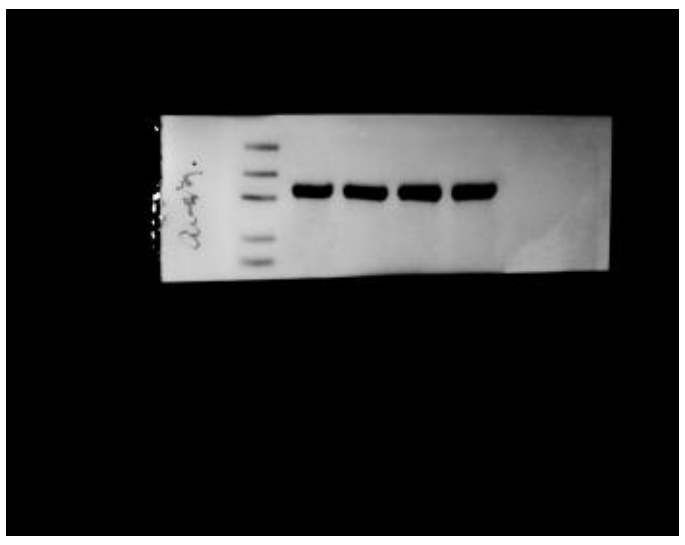

Fig. S1A

RanBP3 (70KDa) β-actin (42KDa)

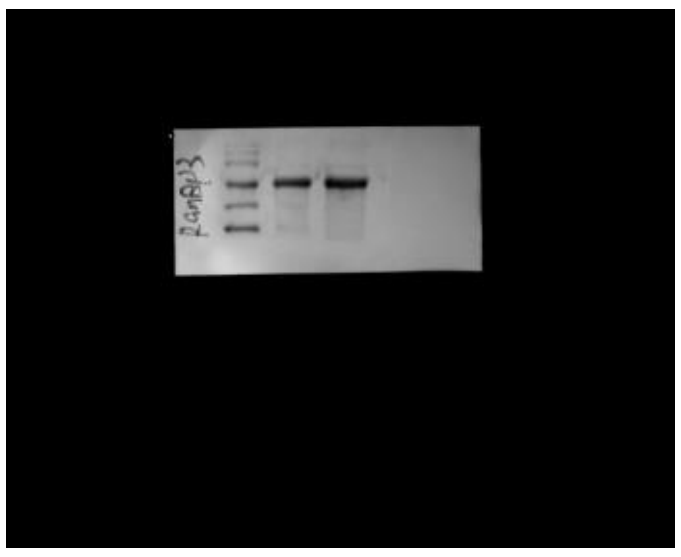

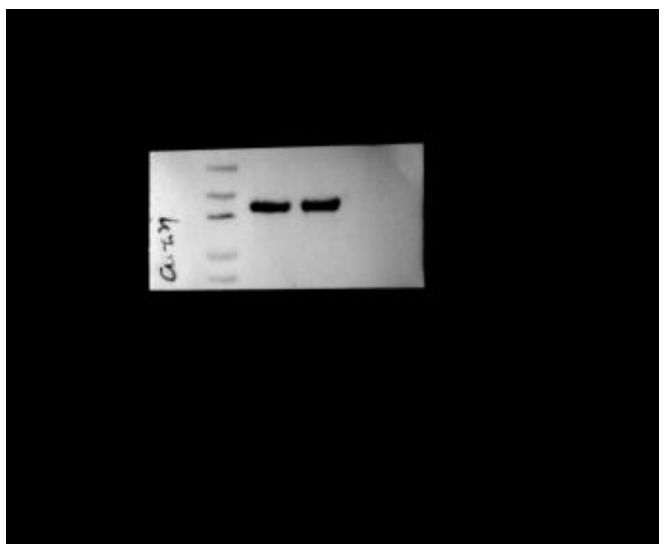

Fig. S1B

RanBP3 (70KDa) β-actin (42KDa)

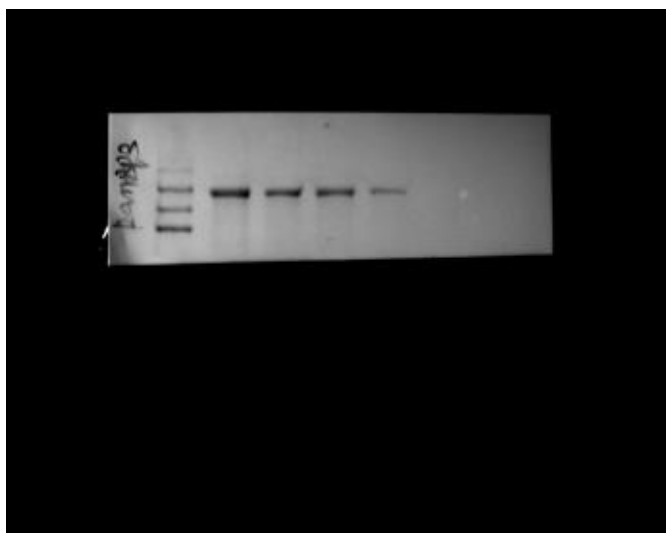

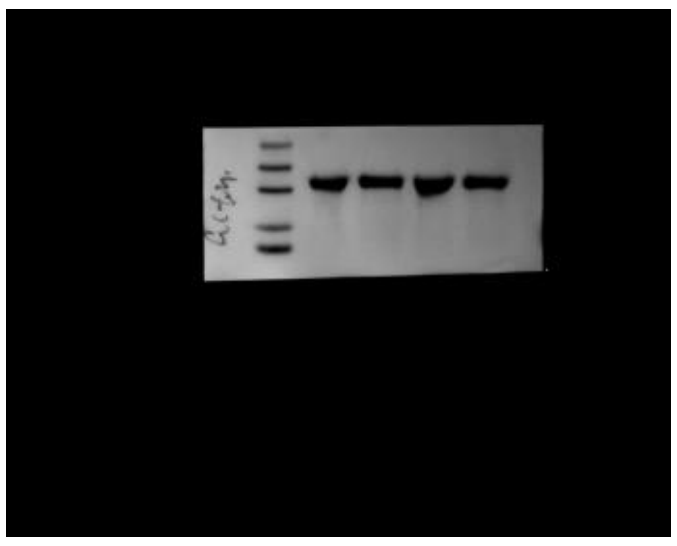

Fig. S1C

CCAR2 (130KDa) β-actin (42KDa)

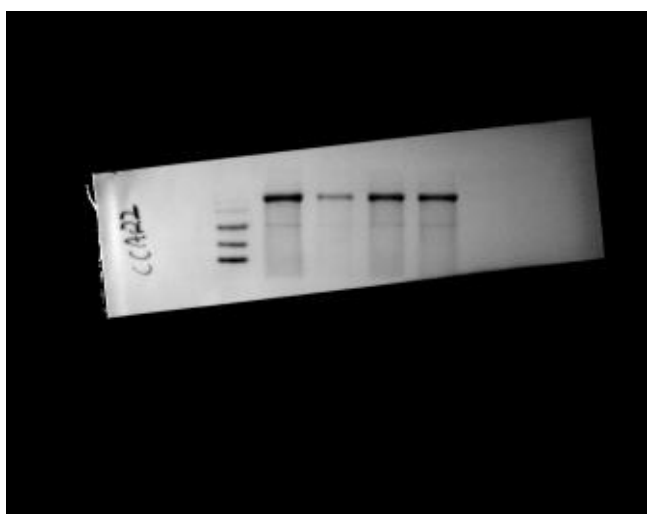

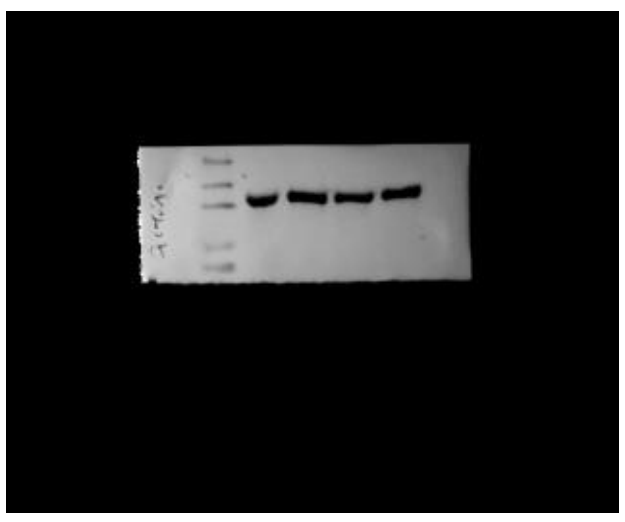

Fig. S1D

SIRT1 (120KDa)

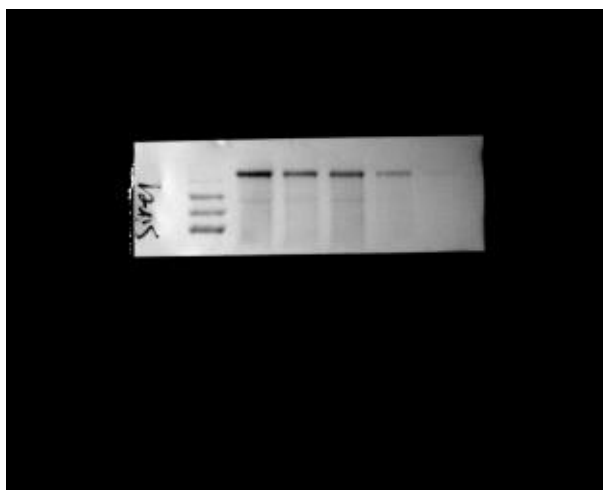

β-actin (42KDa)

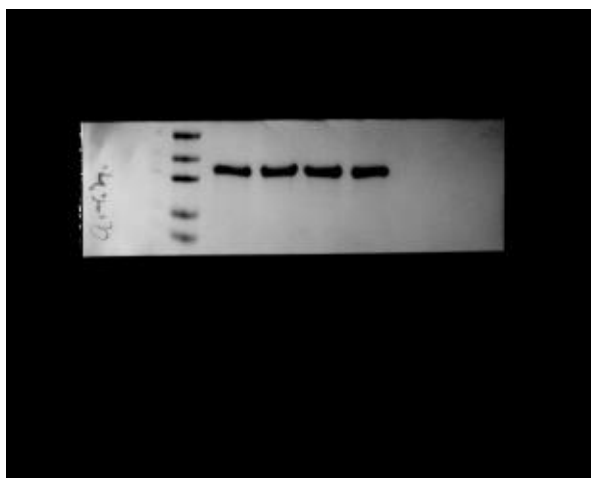

Supplement: Supplementary file 1 [file 41598_2026_55696_MOESM1_ESM.pdf]
